# An Increase in the Prevalence of Clinically Relevant Resistance-Associated Substitutions in Four Direct-Acting Antiviral Regimens: A Study Using GenBank HCV Sequences

**DOI:** 10.3390/pathogens13080674

**Published:** 2024-08-09

**Authors:** Roaa Khalil, Kholoud Al-Mahzoum, Muna Barakat, Malik Sallam

**Affiliations:** 1 Department of Pathology, Microbiology and Forensic Medicine, School of Medicine, The University of Jordan, Amman 11942, Jordan; 2 Department of Clinical Pharmacy and Therapeutics, Faculty of Pharmacy, Applied Science Private University, Amman 11931, Jordan; 3 Department of Clinical Laboratories and Forensic Medicine, Jordan University Hospital, Amman 11942, Jordan

**Keywords:** resistance, therapy, treatment response, treatment as prevention, HCV elimination

## Abstract

Direct-acting antivirals (DAAs) revolutionized the therapeutics of chronic hepatitis C. The emergence and transmission of HCV variants with resistance-associated substitutions (RASs) can undermine HCV treatment. This study aimed to assess the prevalence and temporal trends of RASs in HCV, with a particular focus on clinically relevant RASs (cr-RASs). Near-complete HCV GenBank sequences archived in the Los Alamos HCV Database were analyzed. The study period was divided into two phases: before 2011 and from 2011 onward. Identification of RASs across three DAA classes (NS3, NS5A, and NS5B inhibitors) was based on the 2020 EASL guidelines. The AASLD-IDSA recommendations were used to identify cr-RASs for three HCV genotypes/subtypes (1a, 1b, and 3) and four DAA regimens: ledipasvir/sofosbuvir; elbasvir/grazoprevir; sofosbuvir/velpatasvir; and glecaprevir/pibrentasvir. The final HCV dataset comprised 3443 sequences, and the prevalence of RASs was 50.4%, 60.2%, and 25.3% in NS3, NS5A, and NS5B, respectively. In subtype 1a, resistance to ledipasvir/sofosbuvir was 32.8%, while resistance to elbasvir/grazoprevir was 33.0%. For genotype 3, resistance to sofosbuvir/velpatasvir and glecaprevir/pibrentasvir was 4.2% and 24.9%, respectively. A significant increase in cr-RASs was observed across the two study phases as follows: for ledipasvir/sofosbuvir in subtype 1a, cr-RASs increased from 30.2% to 35.8% (*p* = 0.019); for elbasvir/grazoprevir in subtype 1a, cr-RASs increased from 30.4% to 36.1% (*p* = 0.018); In subtype 1b, neither ledipasvir/sofosbuvir nor elbasvir/grazoprevir showed any cr-RASs in the first phase, but both were present at a prevalence of 6.5% in the second phase (*p* < 0.001); for sofosbuvir/velpatasvir in genotype 3, cr-RASs increased from 0.9% to 5.2% (*p* = 0.006); and for glecaprevir/pibrentasvir, cr-RASs increased from 12.0% to 29.1% (*p* < 0.001). The rising prevalence of HCV RASs and cr-RASs was discernible. This highlights the necessity for ongoing surveillance and adaptation of novel therapeutics to manage HCV resistance effectively. Updating the clinical guidelines and treatment regimens is recommended to counteract the evolving HCV resistance to DAAs.

## 1. Introduction

Hepatitis C virus (HCV) infection continues to represent a formidable public health challenge, with an estimated 50 million people having chronic HCV globally [1,2,3]. Additionally, about one million new HCV infections are estimated to occur each year, with an estimated mortality of slightly less than a quarter of a million in 2022 [1]. The chronic nature of HCV infection in a majority of infected individuals can lead to grave outcomes, including cirrhosis and hepatocellular carcinoma (HCC), contributing significantly to global morbidity and mortality [4,5,6,7].

From 2011 onwards, the approval and widespread use of different direct-acting antivirals (DAAs) represented a critical breakthrough in the treatment of HCV [8,9]. The DAAs were reported to offer cure rates exceeding 90% across various HCV genotypes (e.g., genotypes 1 and 4) [10,11]. Nevertheless, lower cure rates (66–80%) were reported for genotype 3, especially among cirrhotic patients, justifying its description as the possible “last stand” for HCV or alternatively “the new HCV villain” [12,13,14,15]. In addition to the high cure rates, the use of DAAs reduced the duration of treatment needed to achieve sustained virologic response (SVR) to 12 weeks [16,17]. Importantly, the introduction and use of DAAs enhanced the implementation of the treatment as prevention (TasP) strategy, significantly reducing HCV transmission (e.g., among injection drug users (IDUs) in Europe) [18,19]. This was highlighted by van Santen et al. in a multinational cohort study that showed a decrease in the pooled incidence of HCV from 0.91 per 100 person-years in 2015 to 0.41 per 100 person-years in 2019 [20]. The TasP strategy is critical to achieving the World Health Organization (WHO) goal of HCV elimination as a public health threat by 2030, considering the current lack of an effective HCV vaccine and the challenges to achieving such a vaccine in the near future [21,22,23].

The current therapeutic strategies for HCV employ a combination of DAAs that target specific viral proteins, namely the NS3 (protease), NS5A (a critical component of the HCV replication complex), and NS5B (the HCV RNA-dependent RNA polymerase (RdRp)) [3,24]. The HCV treatment regimens are tailored based on various factors such as the HCV genotype and viral load, prior treatment, co-infection with human immunodeficiency virus (HIV), and the presence of liver cirrhosis, among other factors [25,26,27].

Despite the remarkable success of DAAs in reducing the HCV burden, the emergence of resistance-associated substitutions (RASs) represents an increasing challenge [28,29,30]. The rapid evolution of HCV driven by error-prone RdRp facilitates the emergence of RASs with subsequent selective advantage under drug pressure [31,32]. The RASs are defined as the amino acid substitutions that reduce the potency of a DAA in vitro or in vivo, with the possibility of treatment failure [30,33,34]. Importantly, the RASs vary based on the specific HCV genotypes/subtypes due to the high genetic diversity of HCV, which leads to different reference sequences (wild types) for different HCV genotypes/subtypes [3,35].

The characterization of RASs initially emerged from studies involving patients treated with DAAs [34,36]. It is important to emphasize that the detection of an RAS per se is not necessarily an indication of resistance to DAAs. Instead, the likelihood of a DAA selecting for an RAS conferring resistance is influenced by the genetic barrier to resistance, the level of drug exposure, and the replicative fitness of the HCV variant [37]. It is also important to highlight that the genetic barriers to resistance vary across different DAAs and are further influenced by variations in HCV genotypes/subtypes [38,39,40].

In detail, the genetic barrier to resistance varies significantly across different DAAs [41]. For example, DAAs with a low genetic barrier, such as some NS5A inhibitors, typically require one or two mutations for the HCV to develop drug resistance [3]. In contrast, DAAs with a medium genetic barrier, such as certain protease inhibitors, necessitate multiple mutations [42]. High genetic barrier DAAs, including NS5B polymerase inhibitors, require several mutations, reducing the likelihood of resistance development [43]. Additionally, the genetic barrier is influenced by variations in HCV genetic composition. For example, HCV of subtype 1a generally develops resistance more rapidly than HCV isolate of subtype 1b [44]. Genotype 3 also exhibits a higher propensity for resistance to some DAAs [12]. Other critical factors include the level of drug exposure and the replicative fitness of HCV variants [41]. Suboptimal DAA levels can lead to the selection of resistant variants, while variants with high replicative fitness can persist and dominate despite DAA therapy [45]. These considerations are vital for optimizing HCV treatment regimens in diverse patient populations [46].

The evaluation of the real-world clinical impact of RASs has been limited and has shown variable results [47,48,49,50,51]. In these studies, the presence of RASs was infrequently associated with treatment failure; nevertheless, RASs were associated with treatment failure more commonly in patients with cirrhosis [52]. Notably, the presence of baseline NS5A RASs is among the factors to be considered for therapy with NS5A inhibitors—at least for some regimens—due to the relatively low genetic barrier for resistance [27,38,41].

An important concept should be clarified at this point in relation to the distinction between RASs and clinically relevant RASs (cr-RASs) [3,26]. The cr-RASs can be defined as the mutations that have been empirically shown to reduce the effectiveness of DAAs, manifested in a high-fold change in potency [26,41,53]. Thus, the characterization of cr-RAS can be crucial for optimizing HCV treatment, as their presence can necessitate adjustments in therapeutic approaches, such as the selection of more potent DAA combinations or the inclusion of additional therapeutic agents to overcome resistance [41,52].

Among the available DAAs in formulation, four regimens—ledipasvir/sofosbuvir (Harvoni), elbasvir/grazoprevir (Zepatier), sofosbuvir/velpatasvir (Epclusa), and glecaprevir/pibrentasvir (Mavyret)—have been distinguished for their broad genotypic effectiveness and high barrier to resistance [3,54,55]. These regimens offer potent therapeutic options to accommodate a wide range of patient demographics and virological profiles. Therefore, monitoring the prevalence and trends of cr-RASs among these regimens is essential to guide clinical and public health strategies aimed at HCV management, prevention, and possible elimination. This surveillance can enable the prediction of potential treatment outcomes and proactively adapt new therapeutic approaches. Moreover, understanding these trends helps in anticipating future challenges in the fight against HCV, which would help to ensure that HCV therapeutic regimens remain effective and that the goals of reducing HCV disease burden and achieving viral elimination by 2030 are met. Therefore, this study aimed to assess the prevalence of RASs, cr-RASs, and their temporal trend using publicly available HCV GenBank sequences across the commonly found genotypes/subtypes.

## 2. Materials and Methods

### 2.1. Study Design

This study was based on a detailed analysis of near-complete HCV genomes, which were obtained from the GenBank sequences archived in the Los Alamos HCV Database (LAHCVDB) [56,57,58]. The selection of near-complete genomes aimed to ensure highly accurate HCV genotype/subtype assignment, which was essential for subsequent analysis of RAS prevalence per HCV genotype/subtype.

### 2.2. Sequence Retrieval, Selection, Alignment, and Subtyping

The initial phase of sequence retrieval started by downloading the full HCV genomes available at LAHCVDB. A total of 5004 HCV sequences in FASTA format were retrieved. The metadata for each sequence was downloaded as well, including data on genotype/subtype, country of sequence collection (if available), year of sequence collection, and accession number. These metadata were standardized in the FASTA headers using FaBox version 1.61, an online fasta sequence toolbox [59]. Sequence visualization and translation into corresponding amino acid (aa) sequences were conducted in MEGA6 software [60].

To accommodate subsequent temporal analysis, the retrieved HCV sequences that lacked complete metadata regarding the timing of collection were excluded. The remaining sequences underwent alignment against the reference HCV sequence (H77, GenBank accession number: AF009606) using the MAFFT multiple sequence alignment tool [61,62].

Then, the aligned HCV sequences were manually inspected for alignment accuracy (by the first and senior authors independently), and then the alignment was subdivided into three HCV genomic regions based on their positions relative to the H77 reference: NS3 (positions: 3420–5312), NS5A (positions: 6258–7601), and NS5B (positions: 7602–9374). Afterward, the sequences in each dataset were screened to exclude any that contained stop codons or extensive regions of missing nucleotides (greater than 30 bases).

The final dataset included 3488 sequences for NS3, 3487 for NS5A, and 3488 for NS5B, excluding the reference sequence H77. Additional filtration steps were applied to exclude genotype 5 sequences due to their limited numbers (*n* = 5) and any recombinant forms (*n* = 4), and non-1a and non-1b genotype 1 (*n* = 36) due to their limited numbers, which would preclude a reliable assessment of RASs prevalence in these genetic variants of HCV. The final list of 3443 HCV sequences analyzed in this study is available in Supplementary Table S1.

Finally, HCV subtype assignment was confirmed for the final HCV nucleotide sequences using the COMET HCV tool, which rapidly classifies HCV sequences into subtypes [63].

### 2.3. Assessment of RASs in the Final HCV Dataset for the Three DAA Classes

To identify RAS indicating resistance to DAAs across three key classes: NS3 inhibitors, NS5A inhibitors, and NS5B inhibitors, we opted to adhere to the 2020 European Association for the Study of the Liver (EASL) Guidelines for the Treatment of Hepatitis C [25]. This involved analyzing specific aa positions known to influence drug response within the NS3, NS5A, and NS5B protein regions [25]. The positions analyzed were 36, 41, 43, 54, 55, 56, 80, 122, 155, 156, 158, 166, 168, 170, and 175 for NS3; 24, 26, 28, 29, 30, 31, 32, 38, 58, 62, 92, and 93 for NS5A; 150, 159, 206, 282, 316, 320, and 321 for NS5B Nis; and 314, 316, 368, 395, 411, 414, 445, 446, 448, 553, 554, 555, 556, 557, 558, 559, 561, and 565 for NNIs NS5B [25]. A schematic representation of these RASs per HCV subtype/genotype and genomic region is shown in Appendix A.

For the prevalence analysis, we quantified the occurrence of genotype-specific RAS at these positions and assessed their prevalence across the whole HCV genotype/subtype dataset. In the presence of ambiguous aa, denoted by “?” in the alignment, which could result from low sequencing quality or the presence of multiple viral strains within the sample, a conservative approach was adopted; ambiguous amino acids were not classified as resistant. This methodological caution was chosen to avoid overestimation of RAS prevalence.

### 2.4. Assessment of Clinically-Relevant RASs by DAA and HCV Genotypes/Subtypes

As stated in Introduction, it was crucial to distinguish between mere RAS and cr-RAS. Therefore, we put a special focus on the cr-RASs, relying on the “HCV Guidance” by AASLD and IDSA criteria for the identification of cr-RAS as follows [26].

The AASLD-IDSA recommendations for testing, managing, and treating hepatitis C were based on the use data for three genotypes/subtypes (1a, 1b, and 3) and concerning four DAA regimens: ledipasvir/sofosbuvir (Harvoni); elbasvir/grazoprevir (Zepatier); sofosbuvir/velpatasvir (Epclusa); and glecaprevir/pibrentasvir (Mavyret) [26]. It is important to note the absence of specific guidelines for RASs in genotypes 4, 5, and 6 [26].

For Harvoni, the following RASs were deemed cr-RAS for subtype 1a: Q30H/R, L31M/V, or Y93C/H/N; and for subtype 1b: L31V or Y93H. For Zepatier, the following RASs were deemed cr-RAS for subtype 1a: M28A/T, Q30H/R, L31M/V, or Y93C/H/N, and for subtype 1b: Y93H. For Epclusa, the following RAS was deemed cr-RAS for genotype 3: Y93H. For Mavyret, the following RAS was deemed cr-RAS for genotype 3: A30K [26].

### 2.5. Data and Statistical Analysis

Statistical analysis was conducted using IBM SPSS Statistics for Windows, Version 27.0 (IBM Corp., Armonk, NY, USA). To explore the associations between categorical variables, we employed the chi-squared test (χ^2^). To evaluate temporal trends, we utilized the linear-by-linear association test (LBL). The threshold for statistical significance was set at *p* < 0.050. To compute the prevalence, we applied the adjusted Wald method to obtain 95% confidence intervals (CIs). For the effect size of differences in the prevalence of RASs and cr-RASs over the two study phases, we calculated Cohen’s *d* using the following formula: (M2 − M1)/SD pooled, where SD pooled = √ ((SD1^2^ + SD2^2^)/2) and M is the mean calculated based on assigning the presence of RAS as “1” and its absence as “zero”.

Geographically, the analysis categorized countries into six regions: (1) North America, Western Europe, and Australia; (2) Eastern Europe, Russia, and former Soviet Union countries; (3) Asia; (4) the Middle East and North Africa (MENA); (5) Africa; and (6) Latin America and the Caribbean. The study timeline was bifurcated into two phases for temporal analysis: the first phase spanning from 1983 to 2010, before the approval of the first DAA in 2011, and the second phase from 2011 to 2022. This division was based on an attempt to examine the RAS trends before and after the widespread introduction of DAAs.

## 3. Results

### 3.1. HCV Dataset Characteristics

**Figure 1:**
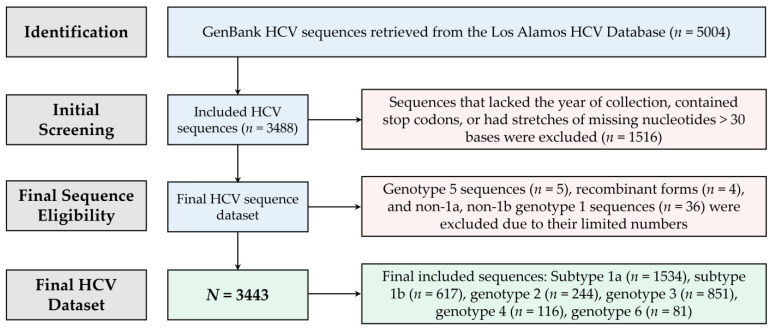
A flowchart of the scheme used to retrieve the final hepatitis C virus (HCV) sequences analyzed in this study.

The final HCV dataset comprised a total of 3443 sequences distributed as follows (Figure 1): subtype 1a (*n* = 1534, 44.6%), subtype 1b (*n* = 617, 17.9%), genotype 2 (*n* = 244, 7.1%), genotype 3 (*n* = 851, 24.7%), genotype 4 (*n* = 116, 3.4%), and genotype 6 (*n* = 81, 2.4%). In the first phase of the study (1983–2010), the number of included HCV sequences was 1650 (47.9%), while in the second phase of the study (2011–2022), the number of HCV sequences was 1793 (52.1%). The number of HCV sequences belonging to genotype 3 increased significantly over the study period, rising from 13.1% in the first phase to 35.4% in the second phase (*p* < 0.001, LBL), while subtypes 1a and 1b decreased significantly from 50.7% and 21.6% in the first phase to 38.9% and 14.6%, respectively.

The majority of included sequences were collected in North America/Western Europe, or Australia, with 2393 sequences (69.5%). The second most common region was Asia, with 413 sequences (12.0%), followed by Africa (*n* = 107, 3.1%), the MENA (*n* = 23, 0.7%), Latin America/the Caribbean (*n* = 20, 0.6%), and only eight sequences were collected in Eastern Europe/Russia (0.2%). A total of 479 HCV sequences lacked information regarding the country/region of collection. The overall prevalence of any RASs in the study sample was 85.8% (95% CI: 84.6–87.0%), with an increase in the RASs prevalence from 78.9% (95% CI: 76.9–80.9%) in the first phase to 92.1% (95% CI: 90.8–93.3%, *p* < 0.001, LBL) in the second phase.

### 3.2. The Prevalence and Trends of NS3 RASs

The overall prevalence of RASs in the NS3 for the included sequences was 50.4% (95% CI: 48.7–52.1%). The majority of these NS3 RASs were observed as a single RAS within the sequences (*n* = 1182, 68.1%), followed by three NS3 RASs (*n* = 342, 19.7%), two RASs (*n* = 202, 11.6%), and only nine sequences harbored four NS3 RASs (0.5%). The full description of the NS3 RASs stratified per HCV GT/SGT is shown in Table 1.

**Figure 2:**
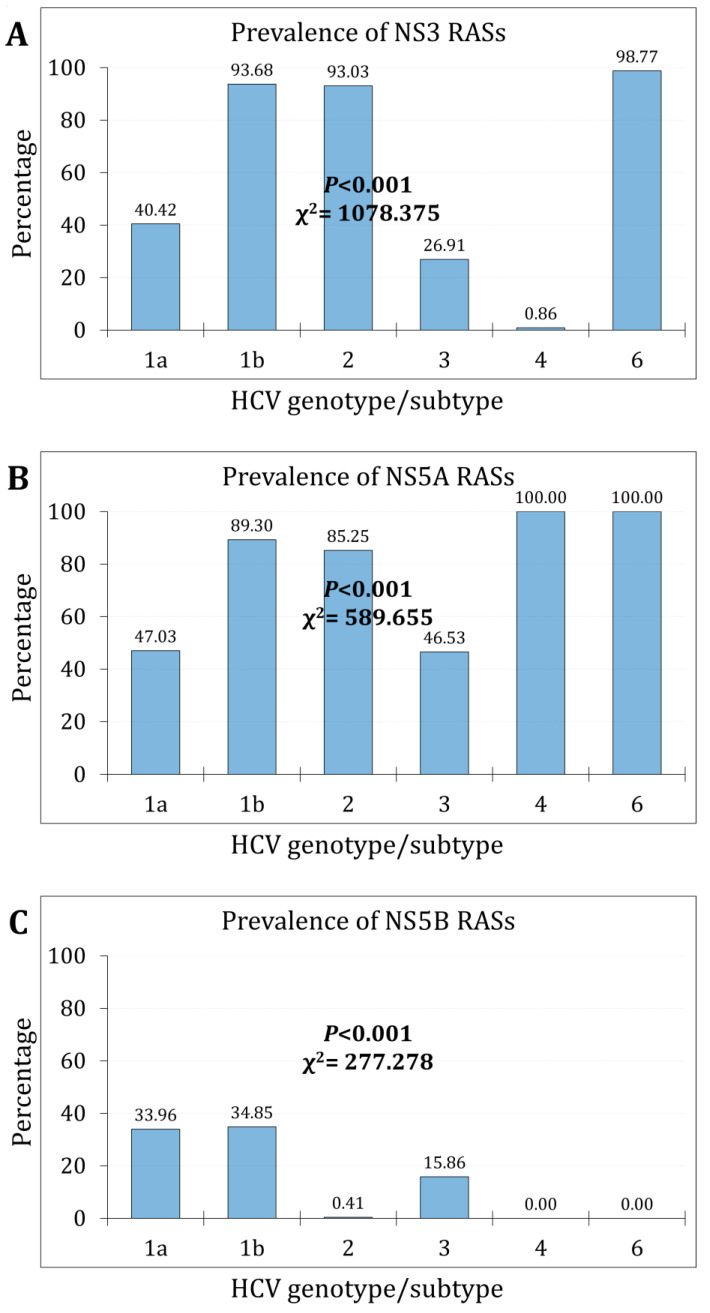
The overall prevalence of RASs per HCV genotype/subtype for NS3 (**A**); NS5A (**B**); and NS5B (**C**).

Stratified per genotype/subtype, the highest prevalence of NS3 RASs was found in genotype 6 (80/81, 98.8%), while the lowest prevalence was found in genotype 4 (1/116, 0.9%, *p* < 0.001, χ^2^ = 1078.375, Figure 2A).

**Figure 3:**
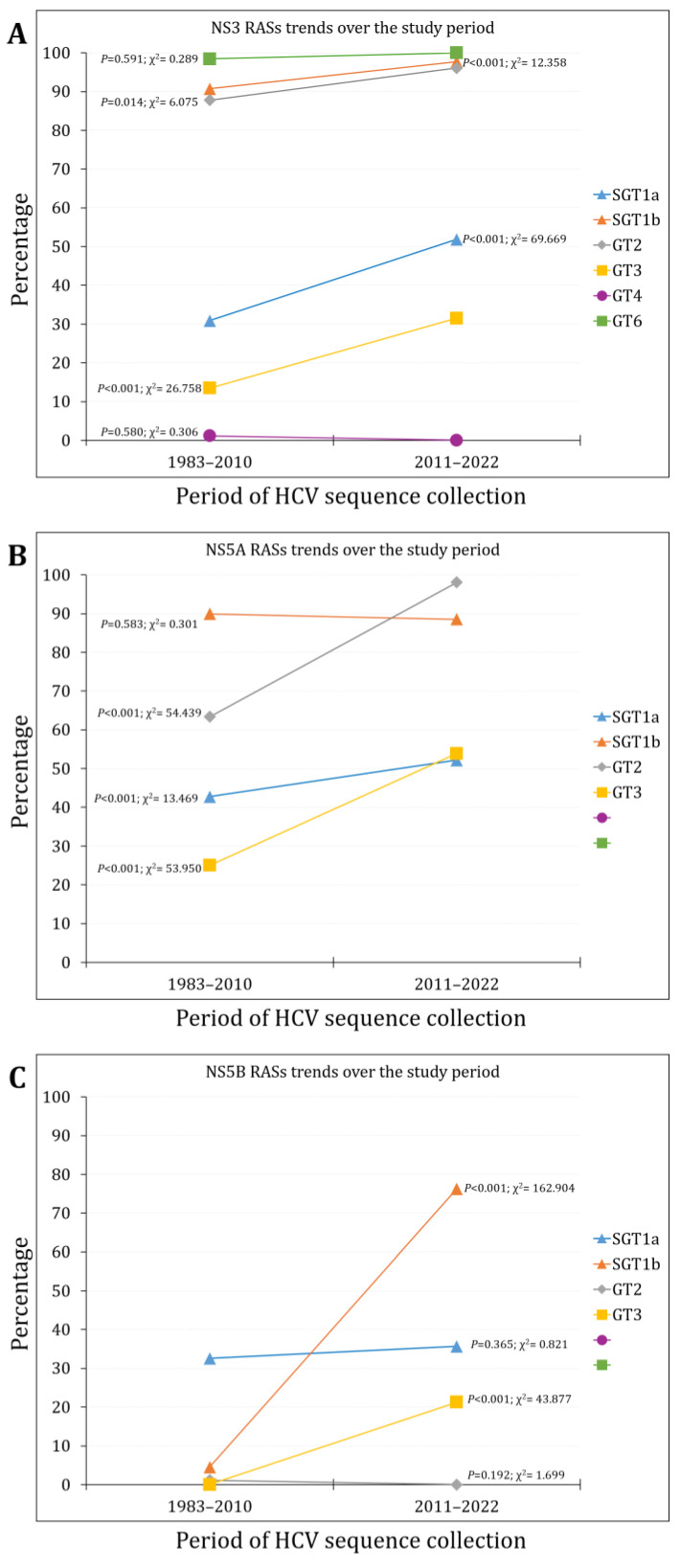
The RASs trends over the study period per HCV genotype/subtype for NS3 (**A**); NS5A (**B**); and NS5B (**C**).

Over the study period, the prevalence of NS3 RASs increased from 45.6% (752/1650) during the first phase to 54.8% (983/1793) during the second phase (*p* < 0.001, χ^2^ = 29.401, Cohen’s *d* = 0.181). The increasing trend was statistically significant for subtype 1a, subtype b, genotype 3 (*p* < 0.001), and genotype 2 (*p* = 0.014, Figure 3A).

**Figure 4:**
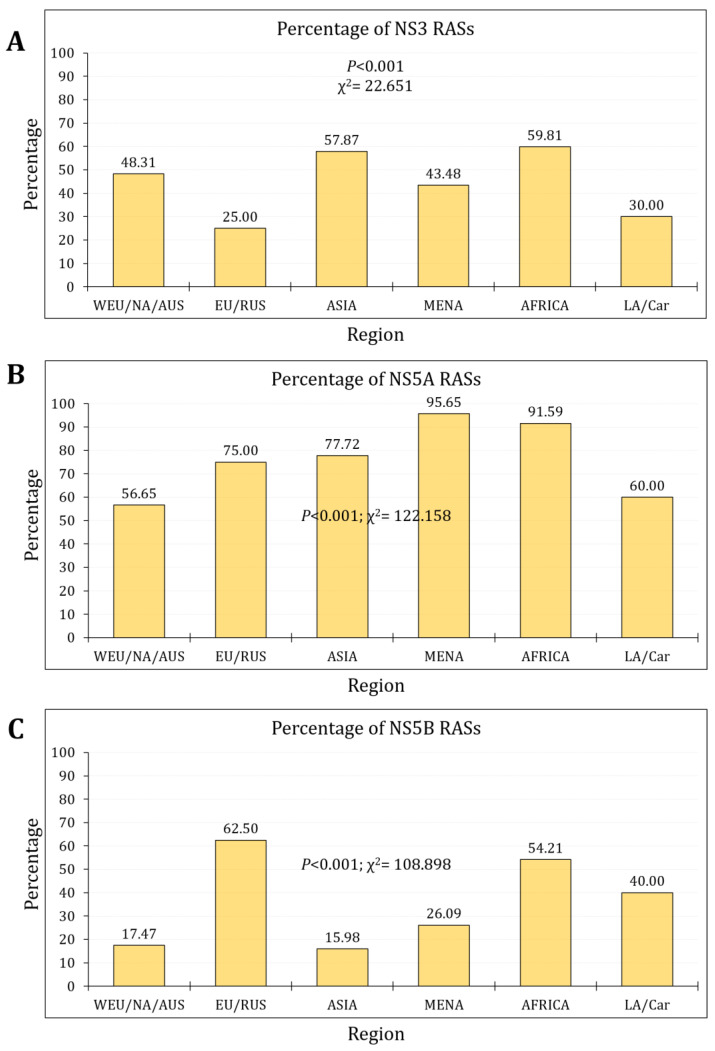
The RASs prevalence per region for NS3 (**A**), NS5A (**B**), and NS5B (**C**). WE/NA/AUS: Western Europe, North America, or Australia; EU/RUS: Eastern Europe, Russia; MENA: Middle East/North Africa; LA/Car: Latin America, the Caribbean.

Per region, the highest percentage of NS3 RASs was observed in Africa (*n* = 64/107, 59.8%), followed by Asia (*n* = 239/413, 57.9%), while the lowest percentage was reported in Eastern Europe/Russia (*n* = 2/8, 25.0%, *p* < 0.001, χ^2^ = 22.651, Figure 4A).

### 3.3. The Prevalence and Trends of NS5A RASs

The overall prevalence of RASs in the NS5A for the included sequences was 60.2% (95% CI: 58.6–61.9%). The vast majority of HCV sequences harboring NS5A RASs contained either three RASs (*n* = 745, 35.9%), a single RAS (*n* = 660, 31.8%), or two RASs (*n* = 633, 30.5%), while 33 sequences contained four RASs (1.6%) and only two sequences contained five RASs (0.1%). The full description of the NS5A RASs stratified per HCV genotype/subtype is shown in Table 2.

At least one NS5A RAS was found in all genotype 4 and genotype 6 sequences, yielding a prevalence rate of 100% in these two genotypes, with genotype 4 comprising the following subtypes: (4d = 62, 53.4%, 4a = 30, 25.9%, and others (4c, 4k, 4m, 4r, and 4v) = 24, 20.7%). On the other hand, genotype 6 comprised the following subtypes: (6a = 28, 34.6%; 6g = 19, 23.5%; 6w = 12, 14.8%; others (6e, 6g, 6k, 6l, 6n, 6v) = 22, 27.2%). Apart from these two genotypes, the highest prevalence of NS5A RASs was observed in subtype 1b (*n* = 551/617, 89.3%), while the lowest prevalence of NS5A RASs was observed in genotype 3 (*n* = 396/851, 46.5%) and subtype 1a (*n* = 721/1533, 47.0%, Figure 2B).

The prevalence of NS5A RASs increased from 57.0% (940/1649) during the first phase to 63.2% (1133/1793) during the second phase (*p* < 0.001, χ^2^ = 13.722, Cohen’s *d* = 0.123). The increasing trend was statistically significant for subtype 1a, genotype 2, and genotype 3 (*p* < 0.001, Figure 3B).

Per region, the highest percentage of NS5A RASs was observed in the MENA and Africa (*n* = 22/23, 95.7% and *n* = 98/107, 91.6%, respectively), while the lowest percentage was reported in Western Europe/North America/Australia (*n* = 1355/2392, 56.6%, *p* < 0.001, χ^2^ = 122.158, Figure 4B).

### 3.4. The Prevalence and Trends of NS5B RASs

The overall prevalence of RASs in the NS5B for the included sequences was 25.3% (95% CI: 23.9–26.8%). The majority of sequences identified with RASs had a single RAS (*n* = 502, 57.6%), followed by sequences with three RASs (*n* = 216, 24.8%), or two RASs (*n* = 149, 17.1%), while only five HCV sequences had four RASs (0.6%). The full description of the NS5B RASs stratified per HCV genotype/subtype is shown in Table 3.

The NS5B RASs were totally absent in all genotype 4 and genotype 6 sequences. Apart from these two genotypes, the highest prevalence of NS5B RASs was observed in subtype 1b (*n* = 215/617, 34.8%) and subtype 1a (*n* = 521/1533, 34.0%), while the lowest prevalence of NS5B RASs was observed in genotype 2 (*n* = 1/244, 0.4%, Figure 2C).

The prevalence of NS5B RASs significantly increased from 17.5% (289/1650) during the first phase to 32.5% (583/1793) during the second phase (*p* < 0.001, χ^2^ = 102.229, Cohen’s *d* = 0.351). The increasing trend was statistically significant only for subtype 1b and genotype 3 (*p* < 0.001, Figure 3C).

Per region, the highest percentage of NS5B RASs was observed in Eastern Europe/Russia (*n* = 5/8, 62.5%) and Africa (*n* = 58/107, 54.2%), while the lowest percentage was reported in Asia (*n* = 66/413, 16.0%, *p* < 0.001, χ^2^ = 108.898, Figure 4C).

### 3.5. The Prevalence of cr-RASs in Four Different DAA Regimens

We proceeded to test the prevalence of cr-RASs as defined in AASLD-IDSA for four currently used DAA regimens, namely Harvoni, Zepatier, Epclusa, and Mavyret.

A total of 502/1533 of subtype 1a had any cr-RAS in relation to Harvoni. A total of 506/1533 of subtype 1a had any cr-RAS in relation to Zepatier. The prevalence of resistance to Harvoni in subtype 1a was 32.8% (95% CI: 30.4–35.1%). The prevalence of resistance to Harvoni and Zepatier in subtype 1b was 2.9% (95% CI: 1.7–4.4%). The prevalence of resistance to Zepatier in subtype 1a was 33.0% (95% CI: 30.7–35.4%), while the prevalence of resistance to Epclusa in genotype 3 was 4.2% (95% CI: 3.0–5.7%). For Mavyret in genotype 3, the prevalence of resistance was 24.9% (95% CI: 22.0–27.8%). The full details of the cr-RASs for the four DAA regimens are presented in Table 4.

**Figure 5:**
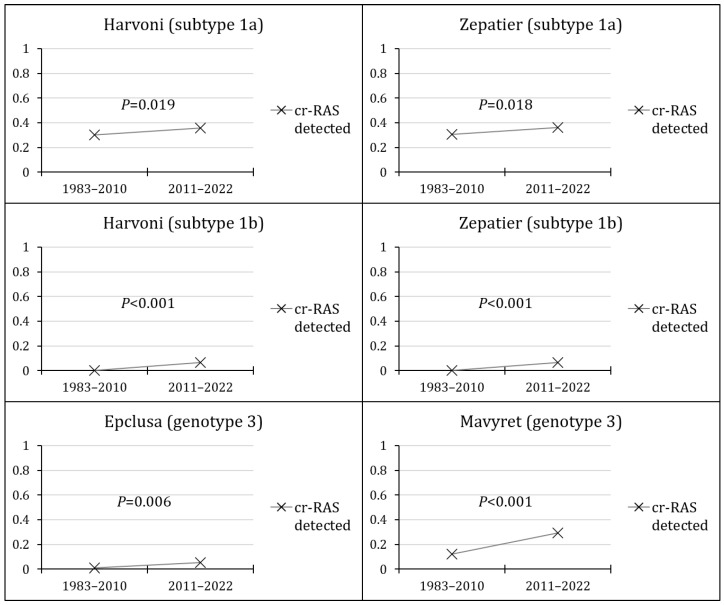
The temporal trends of clinically relevant resistance-associated substitutions (cr-RASs) in four direct-acting antiviral (DAA) regimens.

For the trends of cr-RAS, the prevalence of cr-RAS for Harvoni in subtype 1a increased from 30.2% during the first phase to 35.8% in the second phase (*p* = 0.019, LBL, Cohen’s *d* = 0.128). For Zepatier in subtype 1a, the prevalence of cr-RAS increased from 30.4% during the first phase to 36.1% in the second phase (*p* = 0.018, LBL, Cohen’s *d* = 0.106). In subtype 1b, for both Harvoni and Zepatier, the cr-RAS was totally absent in the first phase and was detected at a prevalence of 6.5% in the second phase (*p* < 0.001, LBL). In genotype 3 and for Epclusa, the prevalence of cr-RAS increased from 0.9% in the first phase to 5.2% in the second phase (*p* = 0.006, LBL, Cohen’s *d* = 0.620). For Mavyret in genotype 3, the prevalence of cr-RAS increased from 12.0% in the first phase to 29.1% in the second phase (*p* < 0.001, Cohen’s *d* = 0.463, LBL, Figure 5).

**Figure 6:**
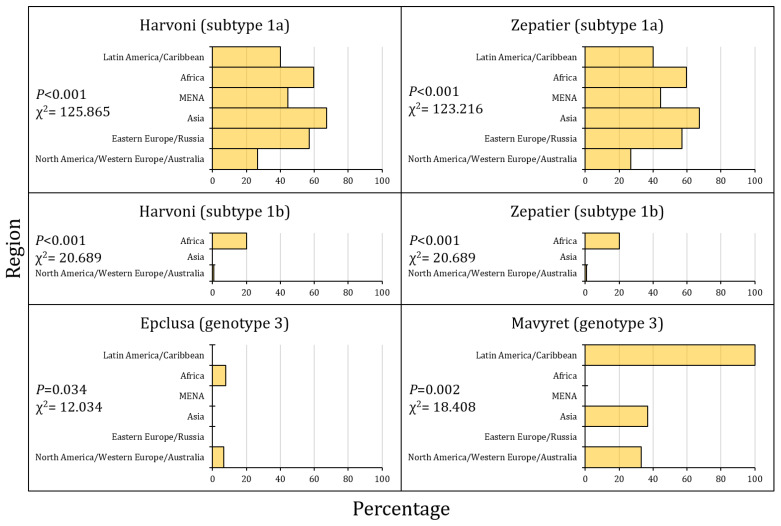
The prevalence of clinically relevant resistance-associated substitutions (cr-RASs) in four direct-acting antiviral (DAA) regimens per region.

**Figure 7:**
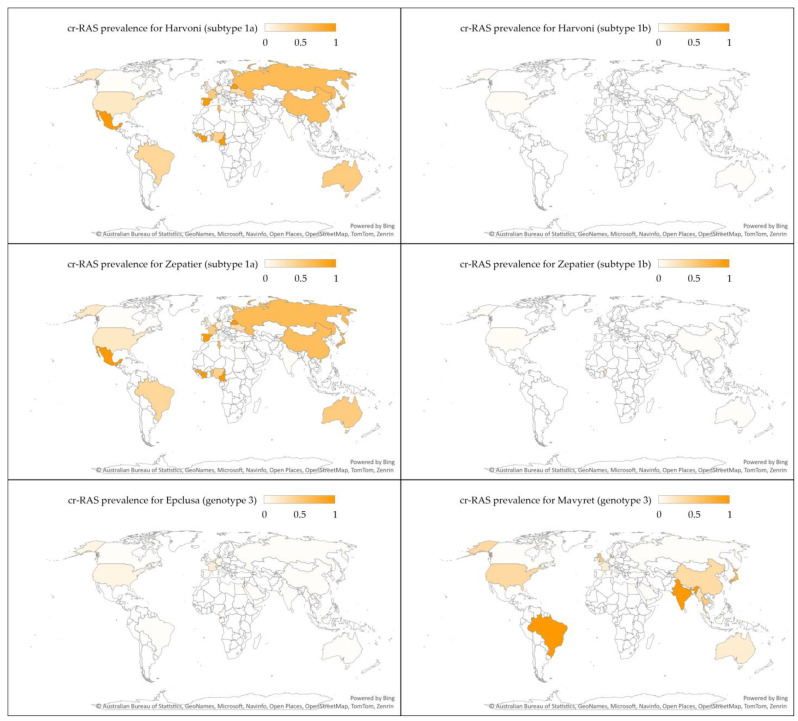
The prevalence of clinically relevant resistance-associated substitutions (cr-RASs) in four direct-acting antiviral (DAA) regimens per country. The map was generated in Microsoft Excel, powered by Bing ©. We are neutral with regard to jurisdictional claims on this map.

Finally, the highest prevalence of cr-RASs was reported in Asia for SGT1a for both Harvoni and Zepatier, in Africa for SGT1b for Harvoni, Zepatier, and Epclusa for GT3, while the highest prevalence of Mavyret cr-RAS for GT3 was reported in Latin America/the Caribbean (Figure 6 and Figure 7).

## 4. Discussions

The findings of this study demonstrated an increasing trend in the prevalence of RASs, including cr-RASs, over the study period. This trend is aligned with the relatively recent widespread adoption of DAAs as a curative therapeutic strategy for chronic hepatitis C and the subsequent selection of drug-resistant HCV variants, which is an unintended consequence of widespread DAA use. However, the detection of RASs during the first phase of the study, prior to the approval of DAAs in 2011, suggested the natural occurrence of RASs, an observation that is consistent with the known rapid evolutionary rate of HCV [32]. The relatively lower prevalence of naturally occurring RASs in the first phase of the study may be attributed to the lower replicative fitness of variants harboring these substitutions [31,65].

Consistent with our finding of a relatively high prevalence of RASs even prior to the widespread use of DAAs, previous studies documented the presence of RASs in DAA-naïve populations [66]. For example, an early study by Paolucci et al. in Italy found that among treatment-naïve individuals, the prevalence of RASs in NS5A and NS5B for subtype 1a was 12.5%, while for subtype 1b, it was significantly higher at 53.3% and 90% for NS5A and NS5B, respectively [67]. A study involving 108 Argentinean DAA-naïve patients reported NS5A and NS5B RASs in 25.8% and 6.3% of the HCV sequences analyzed, respectively [68]. A more recent study in Saint Petersburg found a 12% prevalence of RASs among 42 patients [69].

In this comprehensive study, despite inherent biases in the analyzed HCV sequence data, we utilized 3443 HCV sequences to assess trends in RASs across various HCV genotypes/subtypes and geographic regions from 1983 to 2022. Our findings indicated a clear temporal increase in both RASs and cr-RASs. Specifically, the analysis revealed substantial RAS prevalence rates of 50.4% in NS3, 60.2% in NS5A, and 25.3% in NS5B, suggesting a widespread occurrence of RASs. This trend is supported by the results of an earlier study by Chen et al. in 2016, which reported an overall RAS prevalence of 58.7% across 1459 sequences [70]. Additionally, the common occurrence of NS5A RASs in particular was reported by Kitrinos et al. at a baseline rate of 9.4% in subtypes 1a or 1b patients [71]. The notable escalation in RAS prevalence over time, as demonstrated by the significant rise from the first phase of our study period to the second, can explain the results of this study compared to the aforementioned study on the global prevalence of RASs [70]. This discrepancy can also be attributed to the possible inclusion of HCV sequences collected from DAA-experienced individuals who are more likely to harbor drug-resistant variants [41,72,73,74].

Further evidence of increasing RAS prevalence is provided by a recent study from the SHARED consortium, led by Howe et al. [72] This multinational effort involved collecting HCV sequence-linked metadata from 3355 patients across 22 countries and reported that post-DAA exposure, the frequency of RASs surged from natural baseline levels to 60% in NS3, 80% in NS5A, and 37% in NS5B for specific DAAs, supporting the trends observed in our study. This incremental increase in RASs emphasizes the critical need for ongoing surveillance and novel management strategies to mitigate the impact of RASs on HCV treatment efficacy. Although the prevalence of cr-RASs was relatively low in this study, their potential impact on HCV treatment efficacy cannot be underestimated. The presence of cr-RASs, even at low frequencies, can compromise the effectiveness of DAA therapies in individual patients, particularly those with prior treatment experience or suboptimal adherence. Continuous surveillance to monitor the emergence and spread of cr-RASs is essential for informing treatment guidelines and ensuring that therapeutic regimens remain effective.

On the other hand, the role of these RAS in DAA treatment effectiveness remains to be fully elucidated and might be a contributory factor to consider in cases of treatment failure, depending on the drug regimen [30,49,75,76,77,78]. For example, a systematic review and meta-analysis by Singh et al. reported that baseline RASs decreased the odds of achieving SVR12 in patients with genotype 3 infection [50]. On the contrary, Sarrazin et al. reported a lack of evidence to suggest that known RASs can affect the efficacy of rescue treatments involving multi-target therapies involving sofosbuvir, velpatasvir, and voxilaprevir [49,79].

In this study, the observed disparity in the prevalence of RASs across the NS5A, NS3, and NS5B regions, with NS5A RASs being notably higher, aligns with the biological characteristics of these variants. NS5A is a regulatory protein integral to the HCV replication cycle, assisting in the formation of the replication complex and enhancing replication machinery activity through its interactions with both viral and host proteins [80]. This multifunctional role underlines the persistence of NS5A-resistant variants, which demonstrate significant replicative fitness, thus allowing them to prevail longer than those in NS3 and NS5B [81,82,83,84]. This persistence is further complicated by the fact that NS5A inhibitors, despite their potent antiviral effects, inherently possess a low barrier to resistance [38,85]. This concept is supported by findings from Nitta et al., who documented that specific mutations, namely Y93H and R30Q/A92K in NS5A, were associated with enhanced viral propagation [86,87]. The low resistance barrier of certain DAAs facilitates the rapid emergence of resistant HCV variants, which can replicate in the presence of these drugs [33]. Some mutations enhance replication fitness, allowing these resistant strains to dominate [88]. Additionally, compensatory mutations mitigate fitness costs, promoting HCV variant propagation [89]. This can lead to treatment failure, necessitating regimens that include higher-barrier DAAs to effectively suppress viral replication and prevent resistance.

An important part of this study was the evaluation of the prevalence of cr-RASs for four major DAA regimens—Harvoni, Zepatier, Epclusa, and Mavyret—as defined by the AASLD and the IDSA [26,64]. In contrast to RASs, which are mutations within HCV that have the potential to reduce the effectiveness of DAAs, these cr-RASs are the result of specific mutations that have been demonstrated through clinical studies to significantly affect the efficacy of DAAs, leading to reduced treatment response rates or treatment failures.

Out of 1533 subtype 1a sequences analyzed, 502 showed cr-RASs that affect the efficacy of Harvoni, translating into a prevalence rate of 32.8%. In contrast, only a small fraction (2.9%) of the 617 subtype 1b sequences displayed resistance to both Harvoni and Zepatier. The lower level of resistance in subtype 1b compared to subtype 1a can be explained by previous evidence showing that subtype 1b strains have a higher barrier of resistance compared to subtype 1a strains, with subsequent higher rates of treatment failure [90,91]. The differential prevalence of HCV subtypes 1a and 1b appears to be influenced by the selective pressures of first-generation DAAs such as boceprevir and telaprevir [92,93]. Notably, specific mutations like L31V and Y93H exhibit pronounced resistance in genotype 1a compared to 1b, highlighting distinct genomic and functional barriers to chronic hepatitis C treatment based on the genetic diversity of the HCV isolate [41]. This suggests that these early DAAs have significantly shaped the epidemiological distribution of genotype 1 subtypes, with the prominence of subtypes 1a and 1b in the analyzed datasets in this study.

In clinical trials, the post-treatment resistance of HCV to ledipasvir/sofosbuvir (Harvoni) has been primarily evaluated in patients infected by subtypes 1a or 1b. For example, Wyles et al. showed that virological failure was reported infrequently with Harvoni, occurring in 51 out of 2144 patients (2.4%), and among those who experienced treatment failure, 74.5% harbored detectable mutations associated with resistance to the NS5A inhibitor ledipasvir [73]. Common RASs observed included Q30R/H and/or Y93H/N in subtype 1a and Y93H in subtype 1b, with 35.3% of patients presenting two or more RASs, leading to a significant reduction in ledipasvir susceptibility [73]. Additionally, in a phase 2 study, only one patient with pre-existing ledipasvir resistance developed the sofosbuvir RAS S282T at the point of treatment failure [73]. In this study, the cr-RASs Q30R/H and Y93C/H/N in subtype 1a were reported at rates of 27.8% and 1.9%, respectively, while the cr-RAS Y93H in subtype 1b was observed at a rate of 2.8% of the analyzed sequences, indicating its frequent detection and possible implication on treatment failure associated with Harvoni. On the other hand, the RAS S282T was totally absent in subtypes 1a and 1b sequences analyzed in this study, indicating its infrequent occurrence. In an earlier study, baseline RASs in NS5A and NS5B were reported at a rate of 16% and 2.5%, respectively, with minimal impact on patient responses to ledipasvir/sofosbuvir therapy [94]. Additionally, in the aforementioned clinical trial, the impact of NS5A RASs on outcomes can be overcome by extending the treatment duration to 24 weeks or intensifying therapy [94,95].

Despite being classified as cr-RAS, a Japanese clinical trial found that the Y93H in subtype 1b occurred at a baseline frequency of 17.9% [96]. The study concluded that the presence of baseline NS5A RASs did not affect treatment outcomes in subtype 1b Japanese patients treated with ledipasvir/sofosbuvir (Harvoni) for 12 weeks [96]. On the contrary, another Japanese study that involved 493 patients with subtype 1b infection showed an association between virologic failure and NS5A and NS5B cr-RASs [97]. This suggests the necessity for further research to draw reliable conclusions about the real-world impact of cr-RASs.

In real-world studies, the significant impact of cr-RAS was demonstrated in several studies, as follows: In a cohort of Mongolian patients, Shih-Jer Hsu et al. showed that a higher proportion of the cr-RAS Y93H was the only independent factor associated with treatment failure in subtype 1b infection [98]. A Spanish study analyzed samples from five patients with subtype 1b or genotype 3 infections using deep sequencing before and after failing sofosbuvir/velpatasvir/voxilaprevir therapy [99]. Post-treatment, four patients exhibited the NS5A cr-RAS, Y93H, indicating the selection of HCV RASs that may complicate effective salvage therapies, hinting at the necessity of RAS testing in such a scenario [99].

From a wider perspective, and considering the indeterminate nature of RASs’ impact on DAA therapeutic efficacy, there are valid arguments to show why RASs need to be considered in HCV research and practice. The increasing prevalence of RASs in HCV, as observed in this study, can represent a significant concern since those designated as cr-RASs can lead to treatment failure [29,71,100,101]. Additionally, variants harboring RASs can persist, especially in high-risk populations with lower adherence to therapeutic regimens (e.g., among IDUs) [43,45,102,103]. Consequently, there would be an increased probability of transmission of RASs, with a subsequent negative impact on DAA-naïve individuals [104]. Moreover, the emergence and spread of RASs can limit the effectiveness of existing drugs, which is particularly concerning for patients who have limited treatment options due to previous DAA failures or those with advanced liver disease [48,105,106]. Furthermore, treatment failure negatively impacts patient health and results in increased healthcare costs. This is related to the fact that managing DAA failure involves more complex and prolonged treatment strategies, additional monitoring, and potentially second-line therapies, all of which contribute to higher healthcare expenditures [107]. This is of particular concern in low- and middle-income settings considering the high cost of DAAs, even for generic drugs [108,109,110]. Thus, the emergence and spread of RASs could hinder global efforts for hepatitis C elimination as a public health threat by complicating treatment outcomes and increasing the burden of chronic HCV infections [111].

One important observation of this study was the higher prevalence of RASs in Asia and Africa. This can be attributed to several factors, as follows: In many parts of Asia and Africa, access to the latest DAAs can be limited, and when available, they are often expensive. This leads to inconsistent treatment adherence or the use of suboptimal treatment regimens, which can result in the emergence and persistence of RASs [79]. Additionally, older and less effective DAAs that require longer treatment durations may still be in use in some regions due to economic constraints. These therapies often have a higher risk of developing resistance compared to newer DAAs [72,112]. Furthermore, some regions in Asia and Africa have a higher prevalence of HCV genotypes/subtypes that are naturally more prone to developing certain types of RASs, influencing treatment outcomes [113]. Importantly, the systematic approach for surveillance and monitoring of HCV genetic variants in some regions of Asia and Africa is expected to be less developed. Subsequently, this may delay the detection of RASs and decipher its possible role in treatment failure, allowing for RASs to spread within the population. The lower prevalence of surveillance of RASs in these regions can be inferred from the lower number of available HCV sequences in GenBank, which was illustrated in this study. Lower levels of healthcare quality and the availability of diagnostics for HCV resistance testing can lead to less optimal management of treated individuals, facilitating the persistence and spread of resistant strains.

Finally, it is important to highlight several inevitable caveats that should be taken into consideration when interpreting the study results, as follows: First, this study relied on HCV sequences available from the GenBank and Los Alamos HCV Database, and this might have introduced biases related to the geographic and temporal representation of samples. These databases may not comprehensively reflect the current global diversity of HCV due to the underrepresentation of certain regions or HCV genotypes/subtypes, which was reflected by the dominance of samples from North America and Western Europe. Second, we opted to include near-complete genomes, aiming to enhance the accuracy of genotype/subtype assignments; nevertheless, this approach may have resulted in the inadvertent exclusion of potentially informative partial HCV sequences. Third, the exclusion of sequences without complete metadata, particularly regarding the timing of collection, limited the ability to conduct a more comprehensive analysis by reducing the overall sample size of sequences available for analysis. Fourth, it is important to highlight that the reliance on sequences available from GenBank via different submitting laboratories over a long period of time with varying sequencing technologies and bioinformatics tools carries the inherent limitation of varying sequence quality, particularly in relation to the detection of minor variants. Finally, it is important to stress that the results obtained from the HCV dataset in this study may not be generalizable to all clinical scenarios, particularly in cases where novel or rare subtypes as well as recombinant forms are involved. The clinical relevance of the identified RASs needs to be further validated in prospective clinical studies to ensure their applicability in diverse real-world settings.

## 5. Conclusions

In this study, a comprehensive analysis of 3443 HCV sequences collected from 1983 to 2022 demonstrated a significant increase in the prevalence of RASs and cr-RASs across different HCV genotypes/subtypes and regions. A notably high prevalence of RASs across the three analyzed genomic regions was observed at 50.4%, 60.2%, and 25.3% in NS3, NS5A, and NS5B, respectively. The increasing trend in RASs across genomic regions, genotypes/subtypes, and geographic regions could raise concerns about the ability of these RASs to compromise the efficacy of current therapeutic DAA regimens. This is of particular importance for the increasing prevalence of cr-RASs across four major DAA regimens.

Given these findings, it is crucial to maintain ongoing surveillance of RAS prevalence and develop new antivirals, including novel DAAs, to overcome potential resistance. This approach is essential to preserve the efficacy of current DAAs, reduce HCV transmission, and ultimately achieve the goal of HCV elimination by 2030.

## Figures and Tables

**Table 1:** The full list of NS3 RASs detected in the included sequences stratified per HCV genotype/subtype.

HCV Genotype/Subtype: RASs	*n* (%)	1a (*n* = 1534)	1b (*n* = 617)	2 (*n* = 244)	3 (*n* = 851)	4 (*n* = 116)	6 (*n* = 81)
1a: V36A/C/F/G/L/M 1b: V36A/C/G/L/M 6: V36I	330 (14.8)	121 (7.9)	209 (33.9)	-	-	-	0
1a: T54A/S 1b: T54A/C/G/S	165 (7.7)	33 (2.2)	132 (21.4)	-	-	-	-
1a: V55I 1b: V55A 2: V55A/I	38 (1.6)	14 (0.9)	0	24 (9.8)	-	-	-
1a: Y56H 1b: Y56H/L/F 2: Y56H/F 3: Y56H 4: Y56H 6: Y56H	23 (0.7)	1 (0.1)	9 (1.5)	13 (5.3)	0	0	0
1a: Q80K/L/R 1b: Q80H/K/L/R 3: Q80K/R 4: Q80R 6: L80K/Q	656 (20.5)	377 (24.6)	200 (32.4)	-	0	0	79 (97.5)
1a: S122G/N/R 1b: S122A/D/G/I/N/R/T 6: S122T	216 (9.7)	162 (10.6)	50 (8.1)	-	-	-	4 (4.9)
1a: R155G/I/K/M/Q/S/T/V/W 1b: R155C/G/I/K/L/Q/M/S/T/W 3: R155K 4: R155C/K	11 (0.4)	8 (0.5)	2 (0.3)	-	1 (0.1)	0	-
1a: V158I 1b: V158I	2 (0.1)	1 (0.1)	1 (0.2)	-	-	-	-
3: A166S/T/Y	228 (26.8)	-	-	-	228 (26.8)	-	-
1a: D168A/C/E/F/G/H/I/K/L/N/Q/R/T/V/Y 1b: D168A/C/E/F/G/H/I/K/L/N/Q/T/V/Y 2: D168A/E/F/G/H/N/S/T/V/Y 3: Q168H/K/L/R 4: D168A/E/G/H/T/V 6: D168A/E/G/H/V/Y	421 (12.2)	43 (2.8)	186 (30.1)	190 (77.9)	1 (0.1)	1 (0.9)	0
1a: I/V170T 1b: I/V170A/L/T 6: I170V	7 (0.3)	0	0	-	-	-	7 (8.6)
1b: M175L	551 (89.3)	-	551 (89.3)	-	-	-	-
Sequences with any NS3 RASs	1735 (50.4)	620 (40.4)	578 (93.7)	227 (93.0)	229 (26.9)	1 (0.9)	80 (98.8)

RAS: Resistance-associated substitution; HCV: Hepatitis C virus. The source of these RASs is the 2020 EASL recommendations on the treatment of hepatitis C [25].

**Table 2:** The full list of NS5A RASs in the included sequences stratified per HCV genotype/subtype.

HCV Genotype/Subtype: RASs	*n* (%)	1a (*n* = 1533)	1b (*n* = 617)	2 (*n* = 244)	3 (*n* = 851)	4 (*n* = 116)	6 (*n* = 81)
1a: K24E/Q/R/T 1b: Q24K 2: T24A/S 3: S24F 6: Q24H	1051 (31.6)	502 (32.7)	344 (55.8)	205 (84.0)	0	-	0
1a: M28A/G/S/T/V 1b: L28A/M/T 2: L/F28C/S 3: M28T/K 4: L28M/S/T/V 6: F/L28A/I/L/M/T/V	757 (22.7)	67 (4.4)	522 (84.6)	0	0	84 (72.4)	81 (100.0)
1a: Q30C/D/E/G/H/K/L/N/R/T/Y, del30 1b: R30G/H/P/Q/S 2: L30H/S 3: A30D/E/K/S 4: L30F/G/H/R/S 6: R30E/H/N/S	1105 (33.1)	491 (32.0)	351 (56.9)	3 (1.2)	230 (27.0)	27 (23.3)	3 (3.7)
1a: L31I/F/M/P/V 1b: L31F/I/M/V/W 2: L31I/M/V 3: L31F/I/M/P/V 4: M/L31I/V 6: L31I/M/V	251 (7.5)	6 (0.4)	21 (3.4)	15 (6.1)	190 (22.3)	0	19 (23.5)
1a: H58C/D/L/P/R 1b: P58A/D/L/S/R/T 4: T58A/P/S 6: T58A/G/H/N/S	750 (32.0)	637 (41.6)	3 (0.5)	-	-	76 (65.5)	34 (42.0)
1b: Q/E62D 3: S62L	23 (1.6)	-	6 (1.0)	-	17 (2.0)	-	-
1a: A92K/T 1b: A92E/K/T/V 2: C92R/S/T/W 3: E92K 6: E92T	200 (6.0)	9 (0.6)	189 (30.6)	2 (0.8)	0	-	0
1a: Y93C/F/H/L/N/R/S/T/W 1b: Y93C/H/N/R/S/T 2: Y93F/N/H 3: Y93H/N/S 4: Y93C/H/N/S/R/W 6: T93A/H/N/S	166 (5.0)	41 (2.7)	17 (2.8)	48 (19.7)	52 (6.1)	4 (3.4)	4 (4.9)
Sequences with any NS5A RASs	2073 (62.0)	721 (47.0)	551 (89.3)	208 (85.2)	396 (46.5)	116 (100.0)	81 (100.0)

RAS: Resistance-associated substitution; HCV: Hepatitis C virus. The source of these RASs is the 2020 EASL recommendations on the treatment of hepatitis C [25].

**Table 3:** The full list of NS5B RASs in the included sequences stratified per HCV genotype/subtype.

HCV Genotype/Subtype: RASs	*n* (%)	1a (*n* = 1534)	1b (*n* = 617)	2 (*n* = 244)	3 (*n* = 851)	4 (*n* = 116)	6 (*n* = 81)
**NIs**							
3: A150V	100 (11.8)	-	-	-	100 (11.8)	-	-
1a: L159F 1b: L159F 2: L159F 3: L159F	45 (1.4)	40 (2.6)	3 (0.5)	0	2 (0.2)	-	-
3: K206E	43 (5.1)	-	-	-	43 (5.1)	-	-
1a: S282G/R/T 1b: S282G/R/T 2: S282G/R/T 3: S282G/R/T 4: S282C/G/R/T 6: S282G/R/T	1/3443 (0.03)	0	0	1 (0.4)	0	0	0
1a: C316H/R 1b: C316F/H/N	9 (0.4)	5 (0.3)	4 (0.6)	-	-	-	-
**NNI**							
1a: C316Y 1b: C316H/N/Y/W	5 (0.2)	0	5 (0.8)	-	-	-	-
1a: M414I/T/V 1b: M414I/T/V	8 (0.4)	7 (0.5)	1 (0.2)	-	-	-	-
1b: C445F/Y	203 (32.9)	-	203 (32.9)	-	-	-	-
1a: E446K/Q	418 (27.2)	418 (27.2)	-	-	-	-	-
1a: Y448C/H 1b: Y448C/H	6 (0.3)	4 (0.3)	2 (0.3)	-	-	-	-
1a: A553T/V 1b: A553V	280 (13.0)	85 (5.5)	195 (31.6)	-	-	-	-
1a: G554S 1b: G554S	4 (0.2)	3 (0.2)	1 (0.2)	-	-	-	-
1a: S556G/R 1b: S556G/R	343 (15.9)	142 (9.3)	201 (32.6)	-	-	-	-
1a: D559G/N 1b: D559G/N	2 (0.09)	2 (0.1)	0	-	-	-	-
1a: Y561H/N	1 (0.07)	1 (0.07)	-	-	-	-	-
Sequences with any NS5B RASs	872 (25.3)	521 (34.0)	215 (34.8)	1 (0.4)	135 (15.9)	0	0

RAS: Resistance-associated substitution; HCV: Hepatitis C virus; NI: Nucleoside inhibitor; NNI: non-nucleoside inhibitor. The source of these RASs is the 2020 EASL recommendations on the treatment of hepatitis C [25].

**Table 4:** The clinically-relevant RASs identified for four DAAs.

aa Position in NS5A	28	30	31	93
Regimen				
Harvoni (subtype 1a)	-	Q30H/R	L31M/V	Y93C/H/N
N (%)		426 (27.8)	83 (5.4)	29 (1.9)
Harvoni (subtype 1b)	-	-	L31V	Y93H
N (%)			0	17 (2.8)
Zepatier (subtype 1a)	M28A/T	Q30H/R	L31M/V	Y93C/H/N
N (%)	6 (0.4)	426 (27.8)	83 (5.4)	29 (1.9)
Zepatier (subtype 1b)	-	-	-	Y93H
N (%)				17 (2.8)
Epclusa (genotype 3)	-	-	-	Y93H
N (%)				35 (4.1)
Mavyret (genotype 3)	-	A30K	-	-
N (%)		211 (24.8)		

N: Number; aa: amino acid. The source of these cr-RASs is the AASLD-IDSA HCV Guidance [64].

**Figure A1:**
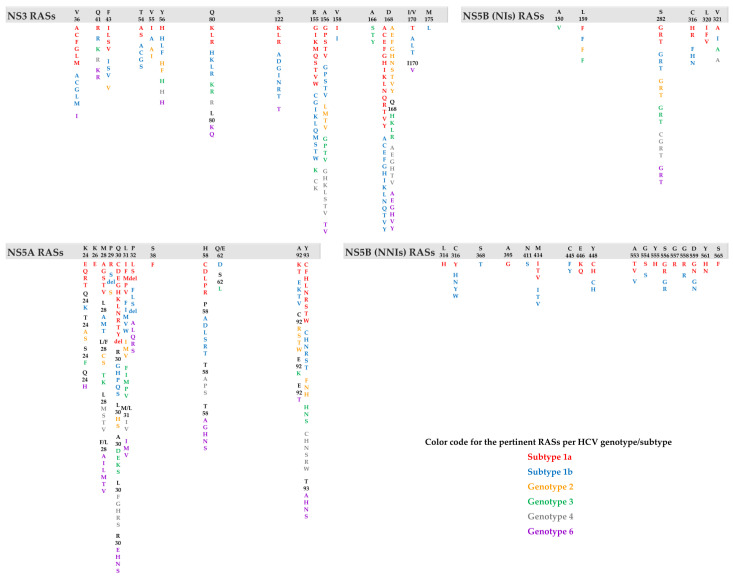
A Schematic of Hepatitis C Virus (HCV) Resistance-Associated Substitutions (RASs) Per HCV Subtype/Genotype and HCV Genomic Region. NI: Nucleoside Inhibitor; NNI: Non-Nucleoside Inhibitor.

## Data Availability

The data that support the findings of this study are available in the supplementary material section of this article.

## Supplementary Materials

The following supporting information can be downloaded at: https://www.mdpi.com/article/10.3390/pathogens13080674/s1, Table S1: The full list of GenBank accession numbers for the HCV sequences analyzed in this study.
