# Supplementary material for: An Increase in the Prevalence of Clinically Relevant Resistance-Associated Substitutions in Four Direct-Acting Antiviral Regimens: A Study Using GenBank HCV Sequences
Source: Pathogens. 2024 Aug 9;13(8):674. doi: 10.3390/pathogens13080674 (PMC11356961; doi:10.3390/pathogens13080674)
Supplement: Supplementary file 1 [file pathogens-13-00674-s001.zip › Supplementary_Table_S1.pdf]

Supplementary Table S1

The full list of GenBank accession numbers for the HCV sequences analyzed in this study

Accession	Accession	Accession	Accession	Accession
AB249644	JX463527	KY620450	MT632148	OK553071
AB429050	JX463528	KY620451	MT632149	OK553072
AB622121	JX463529	KY620452	MT632150	OK553073
AB677527	JX463530	KY620453	MT632151	OK553074
AB677529	JX463532	KY620454	MT632152	OK553075
AB677530	JX463533	KY620455	MT632153	OK553076
AB677531	JX463534	KY620456	MT632154	OK553077
AB677532	JX463535	KY620457	MT632155	OK553078
AB677533	JX463536	KY620458	MT632156	OK553079
AB690460	JX463537	KY620459	MT632157	OK553080
AB690461	JX463538	KY620460	MT632158	OK553081
AB792683	JX463539	KY620461	MT632159	OK553082
AB795432	JX463540	KY620462	MT632160	OK553083
AY587844	JX463541	KY620463	MT632161	OK553084
AY587845	JX463542	KY620464	MT632162	OK553085
AY956463	JX463543	KY620465	MT632163	OK553086
AY956464	JX463544	KY620466	MT632164	OK553087
AY956465	JX463545	KY620467	MT632165	OK553088
AY956467	JX463546	KY620470	MT632167	OK553089
AY956468	JX463547	KY620471	MT632168	OK553090
AY956469	JX463548	KY620472	MT632169	OK553091
D10750	JX463549	KY620473	MT632170	OK553092
D13558	JX463550	KY620474	MT632171	OK553093
D89815	JX463551	KY620475	MT632172	OK553094
DQ430811	JX463552	KY620476	MT632174	OK553095
DQ430813	JX463553	KY620477	MT632175	OK553096
DQ430815	JX463554	KY620480	MT632176	OK553097
DQ430817	JX463555	KY620481	MT632177	OK553098
DQ430819	JX463556	KY620482	MT632178	OL332047
DQ430820	JX463557	KY620484	MT632179	OL332048
DQ889251	JX463558	KY620485	MT632180	OL332049
DQ889252	JX463559	KY620486	MT632181	OL332050
DQ889253	JX463560	KY620487	MT632182	OL332051
DQ889254	JX463561	KY620488	MT632183	OL332052
DQ889255	JX463562	KY620489	MT632184	OL332053
DQ889256	JX463563	KY620490	MT632185	OL332054
DQ889257	JX463564	KY620491	MT632186	OL332055

DQ889258	JX463565	KY620492	MT632187	OL332056
DQ889259	JX463566	KY620493	MT632188	OL332057
DQ889261	JX463567	KY620494	MT632190	OL332058
DQ889262	JX463568	KY620495	MT632191	OL332059
DQ889263	JX463569	KY620496	MT632192	OL332060
DQ889264	JX463570	KY620497	MT632193	OL332061
DQ889265	JX463571	KY620498	MT632194	OL332062
DQ889266	JX463572	KY620499	MT995311	OL332063
DQ889267	JX463573	KY620500	MT995312	OL332064
DQ889268	JX463574	KY620501	MT995313	OL332065
DQ889269	JX463575	KY620502	MT995314	OL332066
DQ889270	JX463576	KY620503	MT995315	OL332067
DQ889271	JX463577	KY620504	MT995316	OL332068
DQ889272	JX463578	KY620505	MT995317	OL332069
DQ889273	JX463579	KY620506	MT995318	OL332070
DQ889274	JX463580	KY620507	MT995319	OL332071
DQ889275	JX463581	KY620508	MT995320	OL332072
DQ889276	JX463582	KY620509	MT995321	OL332073
DQ889277	JX463583	KY620510	MT995322	OL332074
DQ889278	JX463584	KY620511	MT995323	OL332075
DQ889279	JX463585	KY620512	MT995324	OL332076
DQ889280	JX463586	KY620513	MT995325	OL332077
DQ889281	JX463587	KY620514	MT995326	OL332078
DQ889282	JX463588	KY620515	MT995327	OL332079
DQ889283	JX463589	KY620516	MT995328	OL332080
DQ889284	JX463590	KY620517	MT995329	OL332081
DQ889286	JX463591	KY620518	MT995330	OL332082
DQ889287	JX463592	KY620519	MT995331	OL332083
DQ889288	JX463594	KY620520	MT995332	OL332084
DQ889289	JX463595	KY620521	MT995333	OL332085
DQ889290	JX463596	KY620522	MT995334	OL332086
DQ889291	JX463597	KY620523	MT995335	OL332087
DQ889292	JX463598	KY620524	MT995336	OL332088
DQ889293	JX463599	KY620525	MT995337	OL332089
DQ889294	JX463600	KY620526	MT995338	OL332090
DQ889295	JX463601	KY620527	MT995339	OL332091
DQ889296	JX463605	KY620528	MT995340	OL332092
DQ889297	JX463606	KY620529	MT995341	OL332093
DQ889298	JX463607	KY620530	MT995342	OL332094
DQ889299	JX463608	KY620531	MT995343	OL332095
DQ889300	JX463609	KY620532	MT995344	OL332096
DQ988073	JX463610	KY620533	MT995345	OL332097
DQ988074	JX463611	KY620534	MT995346	OL332098

DQ988075	JX463612	KY620535	MT995347	OL332099
DQ988076	JX463613	KY620536	MT995348	OL332100
DQ988077	JX463614	KY620537	MT995349	OL332101
DQ988078	JX463615	KY620538	MT995350	OL332102
DQ988079	JX463616	KY620539	MT995351	OL332103
EF032883	JX463617	KY620540	MT995352	OL332104
EF032884	JX463619	KY620541	MT995353	OL332105
EF032885	JX463620	KY620542	MT995354	OL332106
EF032886	JX463621	KY620543	MT995355	OL332107
EF032887	JX463622	KY620545	MW041294	OL332108
EF032889	JX463623	KY620546	MW041295	OL332109
EF032890	JX463624	KY620548	MW041296	OL332110
EF032891	JX463625	KY620549	MW041297	OL332111
EF032892	JX463626	KY620550	MW041298	OL332112
EF032893	JX463627	KY620551	MW041299	OL332113
EF032894	JX463628	KY620552	MW531222	OL332114
EF032895	JX463629	KY620553	MW629116	OL332115
EF032896	JX463630	KY620554	MW629117	OL332116
EF032898	JX463631	KY620555	MW629118	OL332117
EF032899	JX463632	KY620556	MW629119	OL332118
EF032900	JX463633	KY620557	MW629120	OL332119
EU155213	JX463634	KY620558	MW675788	OL332120
EU155214	JX463635	KY620560	MW675789	OL332121
EU155215	JX463636	KY620563	MW675790	OL332122
EU155216	JX463637	KY620565	MW675791	OL332123
EU155217	JX463638	KY620566	MW675792	OL332124
EU155218	JX463639	KY620567	MW675793	OL332125
EU155219	JX463640	KY620568	MW675794	OL332126
EU155220	JX472005	KY620570	MW675795	OL332127
EU155221	JX472006	KY620571	MW675796	OL332128
EU155222	JX472007	KY620573	MW675797	OL332129
EU155224	JX472008	KY620574	MW675798	OL332130
EU155225	KC155254	KY620575	MW675799	OL332131
EU155226	KC191671	KY620576	MW675800	OL332132
EU155227	KC197226	KY620577	MW689948	OL332133
EU155228	KC197227	KY620579	MW689949	OL332134
EU155229	KC197228	KY620581	MW689951	OL332135
EU155230	KC197230	KY620582	MW689952	OL332136
EU155231	KC197231	KY620583	MW689953	OL332137
EU155232	KC197232	KY620584	MW689955	OL332138
EU155233	KC197233	KY620585	MW689956	OL332139
EU155234	KC197234	KY620586	MW689957	OL332140
EU155235	KC197235	KY620587	MW689958	OL332141

EU155236	KC197236	KY620589	MW689959	OL332142
EU155237	KC197237	KY620590	MW689960	OL332143
EU155238	KC439481	KY620592	MW689961	OL332144
EU155239	KC439482	KY620593	MW689962	OL332145
EU155240	KC439483	KY620594	MW689967	OL332146
EU155241	KC439484	KY620595	MW689968	OL332147
EU155242	KC439485	KY620596	MW689969	OL332148
EU155243	KC439486	KY620597	MW689970	OL332149
EU155244	KC439487	KY620598	MW689971	OL332150
EU155245	KC439488	KY620599	MW689972	OL332151
EU155246	KC439489	KY620600	MW689973	OL332152
EU155247	KC439490	KY620601	MW689974	OL332153
EU155248	KC439491	KY620602	MW689975	OL332154
EU155249	KC439492	KY620603	MW689976	OL332155
EU155250	KC439493	KY620604	MW689977	OL332156
EU155251	KC439494	KY620605	MW689979	OL332157
EU155252	KC439495	KY620606	MW689980	OL332158
EU155253	KC439496	KY620607	MW689981	OL332159
EU155254	KC439497	KY620608	MW689982	OL332160
EU155255	KC439498	KY620609	MW689983	OL332161
EU155256	KC439499	KY620611	MW689984	OL332162
EU155257	KC439500	KY620612	MW689985	OL332163
EU155258	KC439501	KY620613	MW689986	OL332164
EU155259	KC439502	KY620614	MW689987	OL332165
EU155260	KC439503	KY620615	MW689988	OL332166
EU155261	KC439504	KY620616	MW689989	OL332167
EU155262	KC439505	KY620617	MW689990	OL332168
EU155263	KC439506	KY620618	MW689991	OL332169
EU155264	KC439507	KY620619	MW689992	OL332170
EU155265	KC439508	KY620620	MW689993	OL332171
EU155266	KC439509	KY620621	MW689994	OL332172
EU155267	KC439510	KY620622	MW689995	OL332173
EU155268	KC439511	KY620623	MW689996	OL332174
EU155269	KC439512	KY620624	MW689997	OL332179
EU155270	KC439513	KY620625	MW689998	OL332180
EU155271	KC439514	KY620626	MW689999	OL332181
EU155272	KC439515	KY620627	MW690000	OL332182
EU155273	KC439516	KY620628	MW690001	OL332183
EU155274	KC439517	KY620629	MW690002	OL332184
EU155275	KC439518	KY620630	MW690003	OL332185
EU155276	KC439519	KY620631	MW690004	OL332186
EU155277	KC439520	KY620632	MW690005	OL332187
EU155278	KC439521	KY620633	MW690006	OL332188

EU155279	KC439522	KY620634	MW690007	OL332189
EU155280	KC439523	KY620635	MW690008	OL332190
EU155281	KC439524	KY620637	MW690009	OL332191
EU155282	KC439525	KY620639	MW690010	OL332192
EU155283	KC439526	KY620640	MW690011	OL332193
EU155284	KC439527	KY620643	MW690012	OL332194
EU155285	KC844037	KY620644	MW690013	OL332195
EU155286	KC844038	KY620645	MW690014	OL332196
EU155287	KC844039	KY620646	MW690015	OL332197
EU155288	KC844040	KY620648	MW690016	OL332198
EU155289	KC844041	KY620649	MW690017	OL332199
EU155290	KC844042	KY620650	MW690018	OL332200
EU155291	KC844043	KY620651	MW690019	OL332201
EU155292	KC844044	KY620652	MW690020	OL332202
EU155293	KC844045	KY620653	MW715685	OL332203
EU155294	KC844047	KY620654	MW715686	OM222702
EU155295	KC844048	KY620657	MW715687	OM222703
EU155296	KC844049	KY620658	MW715688	OM525854
EU155297	KC844050	KY620659	MW715689	OM525856
EU155298	KC844051	KY620660	MZ161145	OM525857
EU155299	KC844052	KY620661	MZ161146	OM525858
EU155300	KC967476	KY620662	MZ161147	OM525859
EU155301	KC967477	KY620663	MZ161148	OM525860
EU155302	KC967478	KY620664	MZ161149	OM525861
EU155303	KC967479	KY620665	MZ161150	OM525862
EU155304	KF035123	KY620666	MZ161151	OM525863
EU155305	KF035124	KY620667	MZ161153	OM525864
EU155306	KF035125	KY620668	MZ161154	OM525865
EU155307	KF035126	KY620669	MZ161155	OM525866
EU155308	KF035127	KY620670	MZ161156	OM525867
EU155309	KF700370	KY620671	MZ161157	OM525868
EU155310	KJ437295	KY620672	MZ161158	OM525869
EU155311	KJ437300	KY620673	MZ171127	OM525870
EU155312	KJ437342	KY620674	MZ171128	OM525872
EU155313	KM043272	KY620675	MZ171129	OM525873
EU155314	KM043273	KY620676	MZ171130	OM525874
EU155315	KM043274	KY620677	MZ171131	OM525875
EU155316	KM043275	KY620678	MZ171132	OM525876
EU155317	KM043276	KY620679	MZ325443	OM525877
EU155318	KM043277	KY620680	MZ325444	OM525878
EU155319	KM043278	KY620682	MZ504973	OM525879
EU155320	KM043279	KY620684	MZ504974	OM525880
EU155321	KM043280	KY620685	MZ504975	OM525881

EU155322	KM043281	KY620686	MZ504976	OM525882
EU155323	KM043282	KY620687	MZ541883	OM525883
EU155324	KM043283	KY620689	MZ541884	OM525884
EU155325	KM043284	KY620690	MZ556841	OM525885
EU155326	KM043285	KY620691	MZ556842	OM525886
EU155327	KM102767	KY620692	MZ556843	OM525887
EU155328	KM102768	KY620693	MZ556844	OM525888
EU155329	KM102769	KY620694	MZ556845	OM525890
EU155330	KM102770	KY620695	MZ556846	OM525891
EU155331	KM277568	KY620696	MZ556847	OM525892
EU155332	KM277569	KY620697	MZ556848	OM525893
EU155333	KM277570	KY620698	MZ556849	OM525894
EU155334	KM277571	KY620699	MZ556850	OM525895
EU155335	KM277572	KY620700	MZ556851	OM525896
EU155336	KM277573	KY620701	MZ556852	OM525897
EU155337	KM277574	KY620702	MZ556853	OM525898
EU155338	KM277575	KY620703	MZ556854	OM525899
EU155339	KM277576	KY620705	MZ556855	OM525900
EU155340	KM277577	KY620706	MZ556856	OM525901
EU155341	KM277578	KY620707	MZ556857	OM525902
EU155342	KM277579	KY620708	MZ556858	OM525904
EU155343	KM277580	KY620709	MZ556859	OM525905
EU155344	KM277581	KY620710	MZ556860	OM525906
EU155345	KM587622	KY620711	MZ556861	OM525907
EU155346	KR870888	KY620712	MZ556862	OM525909
EU155347	KT595242	KY620713	MZ556863	OM896881
EU155348	KT734516	KY620714	MZ556864	OM896882
EU155349	KT734517	KY620715	MZ556865	OM896883
EU155350	KT734518	KY620716	MZ556866	OM896884
EU155351	KT734519	KY620717	MZ556867	OM896885
EU155352	KT734520	KY620718	MZ556868	OM896886
EU155353	KT734521	KY620719	MZ556869	OM896887
EU155354	KT734522	KY620720	MZ556870	OM896888
EU155355	KT734523	KY620721	MZ556871	OM896889
EU155356	KT734524	KY620723	MZ556872	OM896890
EU155357	KT734525	KY620724	MZ556873	OM896891
EU155358	KT734526	KY620725	MZ556874	OM896893
EU155359	KT734527	KY620727	MZ556876	OM896894
EU155360	KT734528	KY620728	MZ556877	OM896895
EU155361	KT734529	KY620729	MZ556878	OM896896
EU155362	KT734530	KY620730	MZ556879	OM896897
EU155363	KT734531	KY620731	MZ556880	OM896898
EU155364	KT734532	KY620732	MZ556881	OM896899

EU155365	KT734533	KY620733	MZ556882	OM896900
EU155366	KT734534	KY620734	MZ556883	OM896901
EU155367	KT734535	KY620735	MZ556884	OM896903
EU155368	KT734536	KY620736	MZ556885	OM896904
EU155369	KT734537	KY620737	MZ556886	OM896905
EU155370	KT734538	KY620738	MZ556887	OM896906
EU155371	KT734539	KY620741	MZ556888	OM896907
EU155372	KT734540	KY620742	MZ556889	OM896908
EU155373	KT734541	KY620743	MZ556890	OM896909
EU155374	KT734542	KY620745	MZ556891	OM896910
EU155375	KT734543	KY620746	MZ556892	OM896911
EU155376	KT734544	KY620747	MZ556893	OM896912
EU155377	KT734545	KY620749	MZ556894	OM896913
EU155378	KT734546	KY620750	MZ556895	OM896914
EU155379	KT734548	KY620751	MZ556896	OM896915
EU155380	KT734549	KY620752	MZ556897	OM896916
EU155381	KT734550	KY620753	MZ556898	OM896917
EU158186	KT734551	KY620754	MZ556899	OM896918
EU234061	KT734552	KY620756	MZ556900	OM896919
EU234062	KT734553	KY620757	MZ556901	OM896920
EU234063	KT734554	KY620758	MZ556902	OM896921
EU234064	KT734555	KY620759	MZ556903	OM896923
EU234065	KT734556	KY620760	MZ556904	OM896924
EU239713	KT734557	KY620761	MZ556905	OM896925
EU239714	KT734558	KY620762	MZ556906	OM896926
EU239715	KT734559	KY620763	MZ556907	OM896927
EU239716	KT734560	KY620764	MZ556908	OM896928
EU250017	KT734561	KY620765	MZ556909	OM896929
EU255927	KT734562	KY620766	MZ556910	OM896930
EU255928	KT734563	KY620767	MZ556911	OM896931
EU255929	KT734564	KY620768	MZ556912	OM896932
EU255930	KT734565	KY620769	MZ556913	OM896933
EU255931	KT734566	KY620770	MZ556914	OM896934
EU255932	KT734567	KY620772	MZ556915	OM896935
EU255933	KT734568	KY620773	MZ556916	OM896936
EU255934	KT734569	KY620775	MZ556917	OM896937
EU255935	KT734570	KY620777	MZ556918	OM896939
EU255936	KT734571	KY620778	MZ556919	OM896940
EU255937	KT734572	KY620779	MZ556920	OM896941
EU255938	KT734573	KY620780	MZ556921	OM896942
EU255939	KT734574	KY620781	MZ556922	OM896943
EU255940	KT734575	KY620782	MZ556923	OM896944
EU255941	KT734576	KY620783	MZ556924	OM896945

EU255942	KT734577	KY620784	MZ556925	OM896946
EU255943	KT734578	KY620785	MZ556926	OM896947
EU255944	KT734579	KY620788	MZ556927	OM896948
EU255945	KT734580	KY620789	MZ556928	OM896949
EU255946	KT734581	KY620790	MZ556929	OM896950
EU255947	KT734582	KY620791	MZ556930	OM896951
EU255948	KT734583	KY620792	MZ556931	OM896952
EU255949	KT734584	KY620793	MZ556932	OM896953
EU255950	KT734585	KY620794	MZ556933	OM896954
EU255951	KT734586	KY620795	MZ556934	OM896955
EU255952	KT734587	KY620797	MZ556935	OM896956
EU255953	KT734588	KY620798	MZ556936	OM896957
EU255954	KT734589	KY620799	OK552682	OM896958
EU255955	KT734590	KY620801	OK552683	OM896959
EU255956	KT734591	KY620802	OK552684	OM896960
EU255957	KT734592	KY620803	OK552685	OM896961
EU255958	KT734593	KY620804	OK552686	OM896962
EU255959	KT734594	KY620806	OK552687	OM896963
EU255960	KT734595	KY620808	OK552688	OM896964
EU255962	KT734596	KY620810	OK552689	OM896965
EU255963	KT734597	KY620811	OK552690	OM896966
EU255964	KT734598	KY620812	OK552691	OM896967
EU255965	KT734599	KY620813	OK552692	OM896968
EU255966	KT734600	KY620814	OK552693	OM896969
EU255967	KT734601	KY620816	OK552694	OM896970
EU255968	KT734602	KY620817	OK552695	OM896971
EU255969	KT734603	KY620819	OK552696	OM896972
EU255970	KT734604	KY620820	OK552697	OM896973
EU255971	KT734605	KY620821	OK552698	OM896974
EU255973	KT734608	KY620822	OK552699	OM896975
EU255974	KT734609	KY620823	OK552700	OM896976
EU255975	KT734610	KY620824	OK552701	OM896977
EU255976	KT735178	KY620825	OK552702	OM896978
EU255977	KT735184	KY620826	OK552703	OM896979
EU255978	KT735185	KY620827	OK552704	OM896980
EU255979	KT735186	KY620828	OK552705	OM896981
EU255980	KT983617	KY620829	OK552706	OM896982
EU255981	KU871279	KY620831	OK552707	OM896983
EU255982	KU871280	KY620832	OK552708	OM896984
EU255983	KU871281	KY620833	OK552709	OM896985
EU255984	KU871282	KY620834	OK552710	OM896987
EU255985	KU871283	KY620835	OK552711	OM896988
EU255986	KU871284	KY620836	OK552712	OM896989

EU255987	KU871285	KY620837	OK552713	OM896991
EU255988	KU871286	KY620838	OK552714	OM896993
EU255989	KU871287	KY620840	OK552715	OM896994
EU255990	KU871288	KY620841	OK552716	OM896995
EU255991	KU871289	KY620842	OK552717	OM896996
EU255992	KU871290	KY620843	OK552718	OM896997
EU255994	KU871292	KY620844	OK552719	OM896998
EU255995	KU871293	KY620845	OK552720	OM896999
EU255996	KU871294	KY620846	OK552721	OM897000
EU255997	KU871295	KY620847	OK552722	ON314813
EU255998	KU871296	KY620848	OK552723	ON314815
EU255999	KU871297	KY620849	OK552724	ON314816
EU256000	KU871298	KY620850	OK552725	ON314817
EU256001	KU871299	KY620851	OK552726	ON314818
EU256002	KU871300	KY620852	OK552727	ON314819
EU256003	KU871301	KY620853	OK552728	ON314820
EU256004	KU871302	KY620854	OK552729	ON314821
EU256005	KU871303	KY620855	OK552730	ON314823
EU256006	KU871304	KY620856	OK552731	ON314824
EU256007	KU871305	KY620857	OK552732	ON314825
EU256008	KU871306	KY620858	OK552733	ON314826
EU256009	KU871307	KY620859	OK552734	ON314827
EU256010	KU871308	KY620860	OK552735	ON314828
EU256011	KU871309	KY620862	OK552736	ON630763
EU256012	KU871310	KY620864	OK552737	ON630764
EU256013	KU871311	KY620866	OK552738	ON630765
EU256014	KX621422	KY620867	OK552739	ON630766
EU256015	KX621423	KY620869	OK552740	ON630767
EU256016	KX621424	KY620871	OK552741	ON630768
EU256017	KX621425	KY620872	OK552742	ON630769
EU256018	KX621426	KY620874	OK552743	ON630770
EU256019	KX621427	KY620875	OK552744	ON630771
EU256020	KX621428	KY620876	OK552745	ON630772
EU256021	KX621429	KY620877	OK552746	ON630773
EU256022	KX621430	KY704917	OK552747	ON630774
EU256023	KX621431	KY780122	OK552748	ON630775
EU256024	KX621432	KY780123	OK552749	ON630776
EU256025	KX621433	KY883981	OK552750	ON630777
EU256026	KX621434	KY883982	OK552751	ON630778
EU256027	KX621435	LC209863	OK552752	ON630779
EU256028	KX621436	LC209864	OK552753	ON630780
EU256029	KX621437	LC209865	OK552754	ON630781
EU256030	KX621438	LC209866	OK552755	ON630782

EU256031	KX621439	LC209867	OK552756	ON630783
EU256032	KX621440	LC209868	OK552757	ON630784
EU256033	KX621441	LC209869	OK552758	ON630785
EU256034	KX621442	LC209870	OK552759	ON630786
EU256035	KX621443	LC209871	OK552760	ON630787
EU256036	KX621444	LC209872	OK552761	ON630788
EU256037	KX621445	LC209873	OK552762	ON630789
EU256038	KX621446	LC209875	OK552763	ON630790
EU256039	KX621447	LC209876	OK552764	ON630791
EU256040	KX621448	LC209878	OK552765	ON630792
EU256041	KX621449	LC209879	OK552766	ON630794
EU256042	KX621450	LC209880	OK552767	ON630795
EU256043	KX621451	LC209881	OK552768	ON630796
EU256044	KX621452	LC209884	OK552769	ON630797
EU256045	KX621453	LC209885	OK552770	ON630798
EU256046	KX621454	LC209886	OK552771	ON630799
EU256047	KX621455	LC209887	OK552772	ON630800
EU256048	KX621456	LC368309	OK552773	ON630801
EU256049	KX621457	LC368310	OK552774	ON630802
EU256050	KX621458	LC368311	OK552775	ON630803
EU256051	KX621459	LC368312	OK552776	ON630804
EU256052	KX621460	LC368313	OK552777	ON630805
EU256053	KX621461	LC368314	OK552778	ON630806
EU256055	KX621462	LC368315	OK552779	ON630807
EU256056	KX621463	LC368316	OK552780	ON630808
EU256057	KX621464	LC368318	OK552781	ON630809
EU256058	KX621465	LC368319	OK552782	ON630810
EU256059	KX621466	LC368320	OK552783	ON630811
EU256060	KX621467	LC368321	OK552784	ON630812
EU256061	KX621468	LC368322	OK552785	ON630813
EU256062	KX621469	LC368323	OK552786	ON630814
EU256064	KX621470	LC368324	OK552787	ON630815
EU256065	KX621471	LC368325	OK552788	ON630816
EU256066	KX621472	LC368326	OK552789	ON630817
EU256067	KX621473	LC368327	OK552790	ON630818
EU256068	KX621474	LC368329	OK552791	ON630819
EU256069	KX621475	LC368330	OK552792	ON630820
EU256070	KX621476	LC368331	OK552793	ON630821
EU256071	KX621477	LC368332	OK552794	ON630822
EU256072	KX621478	LC368333	OK552795	ON630823
EU256073	KX621479	LC368334	OK552796	ON630824
EU256074	KX621480	LC368335	OK552797	ON630825
EU256075	KX621481	LC368336	OK552798	ON630826

EU256076	KX621482	LC368337	OK552799	ON630827
EU256077	KX621483	LC368338	OK552800	ON630828
EU256078	KX621484	LC368339	OK552801	ON630829
EU256079	KX621485	LC368340	OK552802	ON630830
EU256080	KX621486	LC368341	OK552803	ON630831
EU256081	KX621487	LC368342	OK552804	ON630832
EU256082	KX621488	LC368343	OK552805	ON630833
EU256083	KX621489	LC368344	OK552806	ON630834
EU256084	KX621490	LC368345	OK552807	ON630835
EU256085	KX621491	LC368346	OK552808	ON630836
EU256086	KX621492	LC368347	OK552809	ON630837
EU256087	KX621494	LC368348	OK552810	ON630838
EU256088	KX621495	LC368349	OK552811	ON630839
EU256089	KX621496	LC368350	OK552812	ON630840
EU256090	KX621498	LC368353	OK552813	ON630842
EU256091	KX621499	LC368355	OK552814	ON630843
EU256092	KX621500	LC368356	OK552815	ON630844
EU256094	KX621501	LC368357	OK552816	ON630845
EU256095	KX621502	LC368358	OK552817	ON630846
EU256097	KX621503	LC368359	OK552818	ON630847
EU256098	KX621504	LC368360	OK552819	ON630848
EU256099	KX621505	LC368361	OK552820	ON630849
EU256100	KX621507	LC368362	OK552821	ON630850
EU256101	KX621508	LC368363	OK552822	ON630851
EU256102	KX621509	LC368364	OK552823	ON630852
EU256103	KX621510	LC368365	OK552824	ON630853
EU256105	KX621511	LC368366	OK552825	ON630854
EU256106	KX621512	LC368368	OK552826	ON630855
EU256107	KX621513	LC368369	OK552827	ON630856
EU260395	KX621514	LC368370	OK552828	ON630857
EU260396	KX621515	LC368371	OK552829	ON630858
EU482831	KX621516	LC368372	OK552830	ON630859
EU482832	KX621517	LC368373	OK552831	ON630860
EU482833	KX621518	LC368374	OK552832	ON630861
EU482834	KX621519	LC368375	OK552833	ON630862
EU482835	KX621520	LC368376	OK552834	ON630863
EU482836	KX621521	LC368378	OK552835	ON630864
EU482837	KX621522	LC368379	OK552836	ON630865
EU482839	KX621523	LC368380	OK552837	ON630866
EU482840	KX621525	LC368381	OK552838	ON630867
EU482841	KX621526	LC368382	OK552839	ON630868
EU482842	KX621527	LC368391	OK552840	ON630869
EU482843	KX621528	LC368393	OK552841	ON630870

EU482844	KX621529	LC368394	OK552842	ON630871
EU482845	KX621530	LC368395	OK552843	ON630872
EU482846	KX621531	LC368396	OK552844	ON630873
EU482847	KX621532	LC368397	OK552845	ON630874
EU482848	KX621533	LC368398	OK552846	ON630875
EU482849	KX621534	LC368400	OK552847	ON630876
EU482850	KX621535	LC368401	OK552848	ON630877
EU482852	KX621536	LC368402	OK552849	ON630878
EU482853	KX621537	LC368403	OK552850	ON630879
EU482854	KX621538	LC368404	OK552851	ON630880
EU482855	KX621539	LC368405	OK552852	ON630881
EU482856	KX621540	LC368406	OK552853	ON630882
EU482857	KX621541	LC368407	OK552854	ON630883
EU482858	KX621542	LC368408	OK552855	ON630884
EU482859	KX621543	LC368409	OK552856	ON630885
EU482860	KX621545	LC368410	OK552857	ON630886
EU482861	KX621546	LC368411	OK552858	ON630887
EU482862	KX621547	LC368413	OK552859	ON630888
EU482863	KX621548	LC368414	OK552860	ON630889
EU482864	KX621549	LC368415	OK552861	ON630890
EU482865	KX621550	LC368416	OK552862	ON630891
EU482866	KX621551	LC368417	OK552863	ON630892
EU482867	KX621552	LC368418	OK552864	ON630893
EU482868	KX621554	LC368419	OK552865	ON630894
EU482869	KX621555	LC368420	OK552866	ON630895
EU482870	KX621556	LC368421	OK552867	ON630896
EU482871	KX766990	LC368422	OK552868	ON630897
EU482872	KX766991	LC368423	OK552869	ON630898
EU482873	KX766992	LC368424	OK552870	ON630899
EU482874	KX766993	LC368425	OK552871	ON630900
EU482875	KX766994	LC368426	OK552872	ON676559
EU482876	KX766995	LC368427	OK552873	OP022902
EU482877	KX766996	LC368428	OK552874	OP022904
EU482878	KX766997	LC368429	OK552875	OP022905
EU482879	KX766998	LC368430	OK552876	OP022906
EU482880	KX766999	LC368431	OK552877	OP022908
EU482881	KX767000	LC368432	OK552878	OP022909
EU482882	KX767001	LC368433	OK552879	OP022910
EU482883	KX767002	LC368434	OK552880	OP022911
EU482884	KX767003	LC368435	OK552881	OP022913
EU482885	KX767004	LC368436	OK552882	OP022915
EU482886	KX767006	LC368437	OK552883	OP022916
EU482887	KX767007	LC368438	OK552884	OP022917

EU482888	KX767008	LC368439	OK552885	OP022918
EU482889	KX767009	LC368440	OK552886	OP022919
EU529676	KX767010	LC368441	OK552887	OP022920
EU529677	KX767011	LC368443	OK552888	OP022921
EU529678	KX767012	LC368445	OK552889	OP555739
EU529679	KX767013	LC368446	OK552890	OP555740
EU529680	KX767014	LC368447	OK552891	OP555741
EU529681	KX767015	LC368448	OK552892	OP555742
EU529682	KX767016	LC368449	OK552893	OP555743
EU569722	KX767017	MG406987	OK552894	OP555744
EU569723	KX767018	MG437050	OK552895	OP555745
EU595697	KX767019	MG878999	OK552896	OP894659
EU595698	KX767020	MG879000	OK552897	OQ832071
EU595699	KX767021	MH742360	OK552898	OQ873159
EU660383	KX767022	MH742361	OK552899	OQ873160
EU660384	KX767023	MH742362	OK552900	OQ873161
EU660385	KX767024	MH742363	OK552901	OQ979408
EU660386	KX767025	MH742364	OK552902	OQ979411
EU660387	KX767026	MH742365	OK552904	OQ979413
EU660388	KX767027	MH742366	OK552905	OR497849
EU687193	KX767028	MH742367	OK552906	OR497853
EU687194	KX767029	MH742368	OK552907	OR497854
EU687195	KX767030	MH742369	OK552908	OR497855
EU781746	KX767031	MH742370	OK552909	OR497856
EU781747	KX767032	MH742371	OK552911	OR497857
EU781749	KX767033	MH743149	OK552912	OR497858
EU781750	KX767034	MH743151	OK552914	OR497860
EU781751	KX767035	MH885469	OK552915	OR497861
EU781752	KX767036	MH921830	OK552916	OR497862
EU781753	KX767037	MK039720	OK552917	OR497863
EU781760	KX767038	MK139015	OK552918	OR497864
EU781763	KX767039	MK327981	OK552920	OR497865
EU781767	KX767040	MK327984	OK552921	OR497866
EU781768	KX767041	MK327985	OK552922	OR497867
EU781770	KX767042	MK327986	OK552923	OR497868
EU781771	KX767043	MK327987	OK552924	OR497869
EU781772	KX767044	MK327988	OK552925	OR497870
EU781773	KX767045	MK327989	OK552926	OR497871
EU781774	KX767046	MK327990	OK552927	OR497872
EU781776	KX767047	MK327991	OK552928	OR497873
EU781777	KX767048	MK327992	OK552929	OR497874
EU781779	KX767049	MK327993	OK552930	OR497875
EU781780	KX767050	MK327996	OK552931	OR497876

EU781781	KX767051	MK327997	OK552932	OR497877
EU781782	KX767054	MK327998	OK552933	OR497878
EU781783	KX767055	MK328001	OK552934	OR497879
EU781785	KX767056	MK328002	OK552935	OR497880
EU781786	KX767057	MK328003	OK552936	OR497881
EU781787	KX767058	MK328005	OK552937	OR497883
EU781788	KX767059	MK328006	OK552938	OR497884
EU781789	KX767060	MK328007	OK552939	OR497885
EU781790	KX767061	MK328010	OK552940	OR497887
EU781793	KX767062	MK328012	OK552941	OR497888
EU781794	KX767063	MK328013	OK552942	OR497889
EU781795	KX767064	MK328014	OK552943	OR497890
EU781796	KX767065	MK328019	OK552944	OR497892
EU781797	KX767066	MK328020	OK552945	OR497894
EU781798	KX767067	MK328021	OK552946	OR497896
EU781799	KX767068	MK328022	OK552947	OR497897
EU781800	KX767069	MK328023	OK552948	OR497898
EU781802	KX767070	MK328026	OK552949	OR497899
EU781803	KX767071	MK468926	OK552950	OR497900
EU781831	KX767072	MK468927	OK552951	OR497904
EU857431	KX767073	MK468928	OK552952	OR497905
EU862824	KX767074	MK527508	OK552953	OR497907
EU862826	KX767075	MK527509	OK552954	OR497909
EU862827	KX767076	MK548363	OK552955	OR497910
EU862828	KX767077	MK548365	OK552956	OR497911
EU862829	KY014622	MK548368	OK552957	OR497912
EU862830	KY120328	MK548369	OK552958	OR497913
EU862831	KY120329	MN164843	OK552959	OR497916
EU862832	KY120330	MN164844	OK552960	OR497919
EU862833	KY120331	MN164845	OK552961	OR497920
EU862835	KY120332	MN164846	OK552962	OR497921
EU862836	KY283130	MN164847	OK552963	OR497923
EU862837	KY348757	MN164849	OK552964	OR497924
EU862838	KY620314	MN164850	OK552966	OR497925
EU862839	KY620316	MN164851	OK552967	OR497929
EU862840	KY620317	MN164852	OK552968	OR497930
EU862841	KY620321	MN164853	OK552969	OR497931
FJ024086	KY620327	MN164854	OK552970	OR497932
FJ024087	KY620328	MN164855	OK552971	OR497933
FJ024274	KY620329	MN164856	OK552972	OR497934
FJ024275	KY620338	MN164857	OK552973	OR497935
FJ024276	KY620339	MN164858	OK552974	OR497936
FJ024277	KY620340	MN164859	OK552975	OR497937

FJ024278	KY620343	MN164863	OK552976	OR497938
FJ024279	KY620346	MN164864	OK552977	OR497939
FJ024280	KY620347	MN164865	OK552978	OR497940
FJ024281	KY620348	MN164866	OK552979	OR497944
FJ024282	KY620351	MN164867	OK552980	OR497946
FJ181999	KY620352	MN164868	OK552981	OR497948
FJ182000	KY620354	MN164869	OK552982	OR497949
FJ182001	KY620356	MN164870	OK552983	OR497950
FJ205867	KY620357	MN164874	OK552984	OR497951
FJ205868	KY620359	MN164875	OK552985	OR497952
FJ205869	KY620362	MN164876	OK552986	OR497953
FJ390394	KY620363	MN240359	OK552987	OR497954
FJ390395	KY620364	MN326295	OK552988	OR497956
FJ390396	KY620365	MN385563	OK552989	OR497959
FJ390397	KY620366	MN385564	OK552990	OR497960
FJ390398	KY620367	MN385583	OK552991	OR497961
FJ390399	KY620368	MN628574	OK552992	OR497962
FJ407092	KY620369	MN628575	OK552993	OR497963
FJ410172	KY620370	MN628577	OK552994	OR497965
FJ478453	KY620371	MN628578	OK552995	OR497966
FN666428	KY620372	MN628579	OK552996	OR497967
FN666429	KY620373	MN628580	OK552997	OR497968
GQ149768	KY620374	MN628581	OK552998	OR497969
GQ275355	KY620375	MN628582	OK552999	OR497970
GQ356200	KY620376	MN628583	OK553000	OR497971
GQ356201	KY620377	MN628584	OK553001	OR497972
GQ356202	KY620378	MN628586	OK553002	OR497973
GQ356203	KY620379	MN628587	OK553003	OR497974
GQ356204	KY620380	MN628588	OK553004	OR497975
GQ356205	KY620381	MN628589	OK553005	OR497977
GQ356206	KY620382	MN628590	OK553006	OR497978
GQ356207	KY620383	MN628591	OK553007	OR497979
GQ356208	KY620384	MN628592	OK553008	OR497982
GQ356209	KY620385	MN628593	OK553009	OR497984
GQ356210	KY620386	MN628594	OK553010	OR497985
GQ356211	KY620387	MN628595	OK553011	OR497986
GQ356212	KY620388	MN628596	OK553012	OR497987
GQ356213	KY620389	MN628597	OK553013	OR497988
GQ356214	KY620390	MN782309	OK553014	OR497989
GQ356215	KY620391	MN782310	OK553015	OR497990
GQ356216	KY620392	MN782311	OK553016	OR497991
GQ356217	KY620393	MN977326	OK553017	OR497992
GU451218	KY620394	MN977327	OK553018	OR497993

GU451219	KY620395	MN977328	OK553019	OR497994
GU451220	KY620396	MN977329	OK553020	OR497995
GU451221	KY620397	MT184369	OK553021	OR497997
GU451222	KY620398	MT184370	OK553022	OR497998
GU451223	KY620399	MT184371	OK553023	OR497999
GU451224	KY620400	MT184372	OK553024	OR498000
HM777358	KY620401	MT184373	OK553025	OR498002
HM777359	KY620402	MT184374	OK553026	OR498004
HQ110091	KY620403	MT185686	OK553027	OR498006
HQ537005	KY620404	MT512570	OK553028	OR498007
HQ537006	KY620405	MT632105	OK553029	OR498008
HQ537007	KY620406	MT632106	OK553031	OR498012
HQ537008	KY620407	MT632107	OK553032	OR498013
HQ537009	KY620408	MT632108	OK553033	OR498015
HQ719473	KY620409	MT632109	OK553034	OR498016
HQ912953	KY620410	MT632110	OK553035	OR498017
HQ912954	KY620412	MT632111	OK553036	OR498018
HQ912955	KY620413	MT632112	OK553037	OR498019
HQ912956	KY620414	MT632113	OK553038	OR498026
HQ912957	KY620415	MT632114	OK553039	OR498028
HQ912958	KY620416	MT632115	OK553040	OR498031
HQ912959	KY620417	MT632116	OK553041	OR498033
JF509175	KY620418	MT632117	OK553042	OR498036
JF509176	KY620419	MT632118	OK553043	OR498039
JF509177	KY620420	MT632119	OK553044	OR498041
JN120912	KY620422	MT632120	OK553045	OR498043
JN714194	KY620423	MT632121	OK553046	OR498044
JQ061779	KY620424	MT632122	OK553047	OR498045
JQ061781	KY620426	MT632123	OK553048	OR498046
JQ061782	KY620427	MT632124	OK553049	OR498047
JQ061783	KY620428	MT632126	OK553050	OR498048
JQ061784	KY620429	MT632128	OK553052	OR498049
JQ061785	KY620430	MT632129	OK553053	OR498050
JQ061786	KY620431	MT632130	OK553054	OR498053
JQ061787	KY620432	MT632131	OK553055	OR498054
JQ065709	KY620433	MT632132	OK553056	OR498055
JQ717254	KY620434	MT632133	OK553057	OR498056
JQ717255	KY620435	MT632134	OK553058	OR498058
JQ717256	KY620436	MT632135	OK553059	OR498059
JQ717257	KY620437	MT632136	OK553060	OR498060
JQ717258	KY620438	MT632137	OK553061	OR498064
JQ717259	KY620440	MT632139	OK553062	OR498065
JQ717260	KY620441	MT632140	OK553063	OR498067

JQ745651	KY620442	MT632141	OK553064	OR498068
JQ914271	KY620443	MT632142	OK553065	OR498069
JQ914272	KY620444	MT632143	OK553066	OR594285
JQ914273	KY620446	MT632144	OK553067	PP092205
JQ914274	KY620447	MT632145	OK553068	PP092206
JX463525	KY620448	MT632146	OK553069	
JX463526	KY620449	MT632147	OK553070	
